# Limiting serine availability during tumor progression promotes muscle wasting in cancer cachexia

**DOI:** 10.1038/s41420-024-02271-1

**Published:** 2024-12-21

**Authors:** Erica Pranzini, Livio Muccillo, Ilaria Nesi, Alice Santi, Caterina Mancini, Giulia Lori, Massimo Genovese, Tiziano Lottini, Giuseppina Comito, Anna Caselli, Annarosa Arcangeli, Lina Sabatino, Vittorio Colantuoni, Maria Letizia Taddei, Paolo Cirri, Paolo Paoli

**Affiliations:** 1 Department of Experimental and Clinical Biomedical Sciences, University of Florence, Florence, Italy; 2 Department of Sciences and Technologies, University of Sannio, Benevento, Italy; 3 AREA Science Park, Padriciano, 99 Trieste, Italy; 4 Department of Experimental and Clinical Medicine, University of Florence, Florence, Italy

**Keywords:** Cancer metabolism, Nutrition disorders

## Abstract

Cancer cachexia is a multifactorial syndrome characterized by a progressive loss of body weight occurring in about 80% of cancer patients, frequently representing the leading cause of death. Dietary intervention is emerging as a promising therapeutic strategy to counteract cancer-induced wasting. Serine is the second most-consumed amino acid (AA) by cancer cells and has emerged to be strictly necessary to preserve skeletal muscle structure and functionality. Here, we demonstrate that decreased serine availability during tumor progression promotes myotubes diameter reduction in vitro and induces muscle wasting in in vivo mice models. By investigating the metabolic crosstalk between colorectal cancer cells and muscle cells, we found that incubating myotubes with conditioned media from tumor cells relying on exogenous serine consumption triggers pronounced myotubes diameter reduction. Accordingly, culturing myotubes in a serine-free medium induces fibers width reduction and suppresses the activation of the AKT-mTORC1 pathway with consequent impairment in protein synthesis, increased protein degradation, and enhanced expression of the muscle atrophy-related genes *Atrogin1* and *MuRF1*. In addition, serine-starved conditions affect myoblast differentiation and mitochondrial oxidative metabolism, finally inducing oxidative stress in myotubes. Consistently, serine dietary deprivation strongly strengthens cancer-associated weight loss and muscle atrophy in mice models. These findings uncover serine consumption by tumor cells as a previously undisclosed driver in cancer cachexia, opening new routes for possible therapeutic approaches.

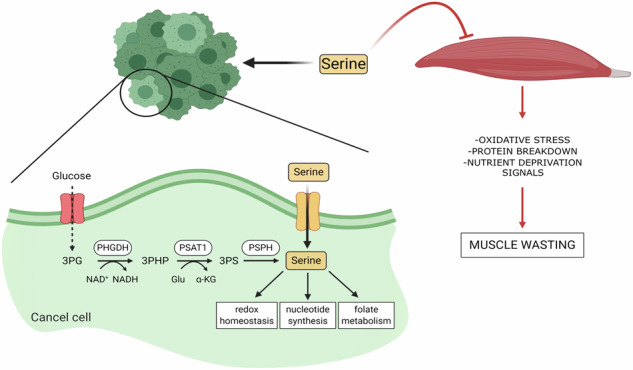

## Introduction

Cachexia is a multifactorial syndrome clinically characterized by a progressive loss of more than 5% of body weight as a result of skeletal muscle atrophy, associated with insulin resistance, anorexia, fatigue, and adipose tissue reduction [1, 2]. Cachexia occurs in about 80% of cancer patients and is associated with poor prognosis, frequently representing the direct cause of patient’s death [3, 4]. Although several therapeutic strategies have been designed to counteract cancer-induced weight wasting, a standard treatment remains undefined [5]. Nevertheless, dietary intervention represents a promising approach [6, 7] in particular, amino acids (AAs) supplementation received special attention [8–10] because of the variations in plasma-free AA levels detected in cancer patients especially those that suffer from cachexia [11, 12].

Serine (Ser) is a non-essential AA that can be generated from the Glycolysis intermediate 3-phosphoGlycerate (3-PG) through the de novo Serine Synthesis Pathway (SSP) by the sequential action of three enzymes PhosphoGlycerate Dehydrogenase (PHGDH), PhosphoSerine Aminotransferase (PSAT1), and PhosphoSerine Phosphatase (PSPH) [13], or it can be obtained by glycine (Gly) through the Serine hydroxymethyl-transferase (SHMT) [14]. Alternatively, it can be taken-up from the extracellular milieu through multiple membrane transporters [15]. Ser is implicated in a broad range of essential cellular functions such as antioxidant defense, one-carbon metabolism, and de novo nucleotide biosynthesis which make it a key nutrient supporting tumor cell proliferation [16]. Coherently, both SSP inhibition [17] and Ser dietary limitation [18, 19] reduce colorectal cancer (CRC) growth both in in vitro and in vivo models, especially when these two approaches are combined [20]. Besides, Ser and Gly are equally necessary to maintain skeletal muscle homeostasis and function under stress conditions such as cachexia-inducing signals [21]. Indeed, increasing evidence underscores an essential role of these two AAs in guaranteeing damaged myofibers repair and maintaining muscle homeostasis after injury [22]. Consistently, Gly or Ser dietary supplementation appears to be functional in preventing tumor-induced muscle wasting and counteracting muscle weakness following anticancer drug exposure in different mice models [23, 24].

In this study, we performed a bioinformatic analysis on two independent murine RNA-Seq datasets to elucidate the metabolic shift underlying cancer-induced cachexia; as ontology breakdown, amino acids metabolism emerged as the primary pathway involved in cachectic processes induced by CRC cancer cells. On the basis of these results, together with studies reporting reduced Ser plasma levels in cachectic CRC-bearing mice [25, 26] and body mass depletion in cancer-bearing patients [27], we hypothesized that tumor-induced alterations in Ser availability could contribute to muscle wasting during cancer cachexia. Our present data demonstrate that Ser consumption by CRC cells contributes to muscle atrophy occurring during cancer cachexia by suppressing the AKT-mTORC1 pathway, thereby affecting protein homeostasis, impairing myoblast differentiation and muscle regeneration, and inducing oxidative stress in myotubes.

## Results

### Transcriptomic analysis of muscle fibers uncovers amino acids metabolism as the main inferred pathway in murine CRC-induced cachexia

**Fig. 1:**
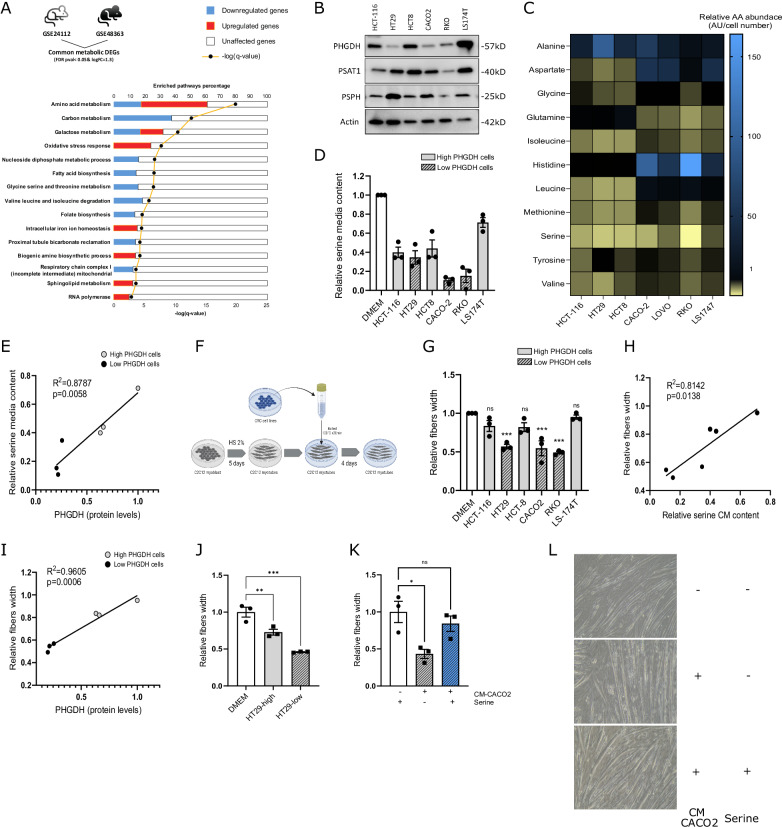
PHGDH expression determines extracellular Ser consumption in cancer cells and correlates with tumor-induced myotubes diameter reduction. **A** Metascape functional enrichment analysis. Common and coherent metabolic DEGs, extracted from GSE24112 and GSE48363, were analyzed for ontology purposes. **B** PHGDH, PSAT1, PSPH protein levels in CRC cell lines. Cell lysates from HCT-116, HT29, HCT8, CACO2, RKO, and LS174T cells were analyzed by western blotting with the anti-PHGDH antibody. An anti-actin antibody was used to ensure equal protein loading. The image is representative of three independent experiments. **C** AA composition of cancer conditioned media (CM). Amino acids content in CM was analyzed by GC-MS on media collected following 48 h of incubation with muscle cells. Data are reported as normalized to serum-free DMEM abundance and are average of three independent experiments. Black label indicates relative value as 1. **D** Ser media content analyzed by GC-MS on media collected following 48 h of incubation with CRC cells. Data are expressed as relative to serum-free DMEM. One-way ANOVA with Sidak’s post hoc test (*n* = 3). Each dot represents a single experiment. **E** PHGDH protein levels-CM Ser content correlation analysis. Pearson correlation between PHGDH protein levels in CRC cell lines and Ser content from corresponding CM. **F** Schematic representation of CM-culturing experiments. **G** Alterations in myotubes width following CM incubation. Relative C2C12 myotubes fibers width following 96 h of incubation with boiled CMs derived from CRC cell lines cultures. CRC cell lines were incubated in serum-free DMEM medium for 48 h before collecting conditioned media (CM) and boiling them for 20 min to remove all protein components. Data are expressed as relative to serum-free DMEM. One-way ANOVA with Sidak’s post hoc test (*n* = 3). Each dot represents a single experiment. **H** Ser media content-myotubes width correlation analysis. Pearson correlation between Ser content in CM from CRC cell lines and C2C12 myotubes width following 96 h of incubation with CM. Data are normalized on values from C2C12 myotubes incubated in normal serum-free DMEM. **I** PHGDH protein levels-myotubes width correlation analysis. Pearson correlation between PHGDH protein levels in CRC cell lines and C2C12 myotubes width following 96 h of incubation with corresponding CM. **J** Alterations in myotubes width following incubation with CM from HT29-high and HT29-low cells. Relative C2C12 myotubes fibers width following 96 h of incubation with boiled CM derived from high PHGDH HT29 and low PHGDH HT29-expressing cells. Data are normalized to width values from C2C12 cells incubated in serum-free DMEM. One-way ANOVA with Dunnett’s post hoc test (*n* = 3). Each dot represents a single experiment. **K** Ser supplementation rescues C2C12 myotubes width reduction under CM incubation. Relative C2C12 myotubes fibers width following 96 h of incubation with boiled CM derived from CACO2 cells supplemented or not with exogenous Ser (0.4 mM). Data are normalized to width values from C2C12 cells incubated in serum-free DMEM. One-way ANOVA with Dunnett’s post hoc test (*n* = 3). Each dot represents a single experiment. **L** Representative microscopic pictures of C2C12 myotubes. C2C12 myotubes were incubated with boiled CM derived from CACO2 cells supplemented or not with exogenous Ser (0.4 mM). Pictures were taken following 96 h of incubation. The image is representative of three independent experiments. Data are represented as mean ± standard error of the mean of at least three independent biological replicates. ns, not significant. *p < 0.05, **p < 0.01, ***p < 0.001.

To investigate the molecular mechanisms underlying CRC-associated cachexia, we performed a bioinformatic analysis by retrieving datasets from GEO of skeletal muscle gene expression in mice with C26 tumor-induced muscle wasting and cachexia (GSE24112 and GSE48363). Among the Differentially Expressed Genes (DEGs) (UP = 2055/DOWN = 2767; UP = 2255/DOWN = 2280, respectively), we selected those commonly and coherently deregulated (908 up- and 754 down-regulated). Pathway analysis was performed by using *metascape* software. The most critical modifications involve: (a) activation of inflammation, with an alteration of the cytokines profile and stress/immune response, as previously reported [28]; (b) downregulation of genes involved in muscle architecture differentiation, with loss of structural integrity and altered proliferation control [29]; (c) upregulation of cellular turnover mechanisms (apoptosis and autophagy) [30, 31]; (d) metabolic rewiring, with mitochondrial respiration inhibition and activation of mechanisms to compensate energy deprivation (Suppl. Figure 1). The limited and controversial information about the metabolic processes involved in the onset and stabilization of cancer-induced cachexia prompted us to perform further analysis filtering DEGs with a list of metabolism-related enzymes and proteins [32]. The results revealed dysregulation of lipid metabolism, including increased lipolysis and altered fatty acid oxidation, alteration in mitochondrial function, impaired energy production, oxidative stress, and, more significantly, variation in amino acids metabolism, particularly in pathways involving Serine, Glycine, and Threonine (Fig. 1A). These results encouraged to investigate more in depth the role of Ser/Gly metabolism in CRC-induced cachexia.

### Extracellular serine consumption by cancer cells participates in cancer-induced myotubes diameter reduction

Several tumor-related systemic alterations are involved in cancer cachexia, including exogenous nutrients release/consumption by cancer cells [33]. To study the impact of cancer metabolism on skeletal muscle homeostasis, we selected six different CRC cell lines previously reported to differ in AA metabolism [34]. The SSP is frequently altered in cancer and PHGDH expression emerged to be very heterogeneous within a single tumor and among various tumor models [35, 36], resulting a strong diversity in exogenous Ser addiction [11, 37]. Selected CRC cell lines differ for SSP enzymes expression (Fig. 1B) and low-expressing PHGDH cells mostly rely on exogenous Ser and have a higher Ser uptake (Suppl. Figure 2A). By analyzing the AA composition of CM by gas-chromatography-mass spectrometry (GC-MS), Ser emerged as the most consumed AA among the analyzed ones (Fig. 1C, D), with an evident correlation between Ser media content and PHGDH expression (Fig. 1E).

We therefore evaluated the effect of incubating C2C12-derived murine myotubes with conditioned media (CM) from cancer cell lines (Fig. 1F). CM were previously boiled to unfold/degrade any protein but preserving metabolites composition. Interestingly, we observed substantial differences in fiber width reduction following 96 h of incubation with CM from the diverse cell lines, indicating that specific cancer cells’ metabolic features participate in the reduction of myotubes diameter. Interestingly, we observed a strong muscle fibers thickness modulation following CM incubation (Fig. 1G). In particular, 96 h of incubation with low-Ser-containing CM induced a more prominent reduction in fiber width than with high-Ser-containing CM. Coherently, 96 h of incubation with CM derived from low-expressing PHGDH cells produced a more prominent reduction in muscle fiber width than following the incubation with high-expressing PHGDH cells-derived CM (Fig. 1G), highlighting a clear inverse correlation between PHGDH cancer cells expression and the reduction in myotubes diameter following CM incubation (Fig. 1H).

Conversely, Gly was not significantly consumed by CRC cells (Fig. 1D, Suppl. Figure 2B). and we did not observe a significative correlation between fibers width and Gly content CM (Suppl. Figure 2C). To further correlate cancer cells’ PHGDH levels and the reduction in myotubes diameter, we incubated C2C12 myotubes with CM from a selected HT29 clone characterized by low PHGDH levels (Suppl. Figure 2D), higher exogenous Ser consumption (Suppl. Figure 2E) and reduced Ser content CM [34]. Coherently, we observed a more prominent reduction in muscle width after 96 h of incubation with CM derived from these cells than from the PHGDH high-expressing companion clone (Fig. 1J). Finally, supplementing low-PHGDH CACO2 cells-derived CM with 0.4 mM Ser fully rescued the CM-induced width decrease in C2C12 myotubes (Fig. 1K, L), demonstrating that a reduced Ser availability contributes to the described myotubes diameter reduction.

Together, these data indicate that Ser consumption by cancer cells inversely correlates with PHGDH status and a decreased Ser content CM results in a more prominent diameter reduction following incubation with myotubes.

### Low-serine plasma levels worsen muscle wasting in CRC in vivo models

**Fig. 2:**
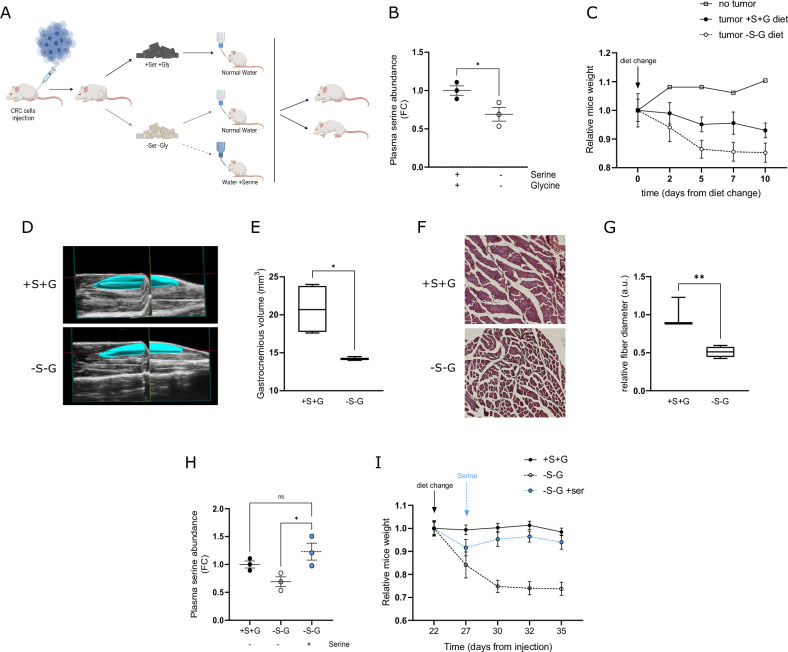
Low Ser plasma levels induce muscle wasting in in vivo CRC models. **A** Workflow for the in vivo assessment of the wasting effect of Ser dietary modulation. CRC cells were injected subcutaneously into the flank of Balb/c mice or Foxn1nu/nu Athymic-Nude mice. Two weeks later, tumor size was assessed by caliper measurement, and mice were randomly divided into experimental groups: control diet, Ser-Gly lacking diet (-S-G diet). Mice were fed with a diet containing Ser and Gly (+S + G) or lacking these two amino acids (-S-G) until the end of the experiment. Ser was added (+Ser) or not in drinking water (20 g/L) 5 days after diet change (*n* = 3). **B** Plasma Ser mice abundance in mice bearing HT29-PHGDH low-derived tumors. Relative Ser levels were quantified by GS-MS analysis in plasma from Foxn1nu/nu Athymic-Nude mice bearing HT29-PHGDH low-derived tumors and fed with +S + G or -S-G diet. Each dot represents a plasma sample derived from a single mouse. Data are represented as mean ± SEM, Student’s t test, **p < 0.01. **C** Evolution of mice weight over the time. Foxn1nu/nu Athymic-Nude mice bearing HT29-low-derived tumors were fed with a +S + G or -S-G two weeks after tumor cells injection (*diet change*). Mice’s weight was assessed every two days until experiment endpoint. Weight values are normalized to the average weight at the day of diet change. Data are represented as mean ± SEM of at least three mice. **D**, **E** Gastrocnemius muscle mass. **D** Representative high-resolution ultrasound images of gastrocnemius muscle of Athymic-Nude mice bearing HT29-low-derived tumors fed with +S + G or -S-G diet. **E** Quantification of gastrocnemius muscle volume of mice as in (**D**) measured by the dedicated in vivo imaging system (Vevo LAZRX photoacoustic imaging). Data are represented as mean ± SEM of three independent measurements, Student’s t test, *p < 0.05. **F** Cross-sectional histological staining of gastrocnemius muscle fiber. Representative images of Hematoxylin and Eosin (H&E) staining of gastrocnemius skeletal muscle collected from Athymic-Nude mice bearing HT29-low-derived tumors fed with +S + G or -S-G diet (magnification ×10). **G** Quantification of gastrocnemius myofiber cross-sectional area. Gastrocnemius skeletal muscles were collected from at least three Athymic-Nude mice bearing HT29-low-derived tumors fed with +S + G or -S-G diet, tissues were stained with H&E, and cross-sectional fibers diameter was qualified by ImageJ. Values are represented as mean ± SEM, Unpaired t-test, **p < 0.01. **H** Ser plasma levels following Ser supplementation in drinking water of mice. Ser levels were quantified by GS-MS analysis in plasma from Foxn1nu/nu Athymic-Nude mice bearing HT29-PHGDH low-derived tumors and fed with +S + G or -S-G diet. Ser was added (+Serine) or not in drinking water (20 g/L) 5 days after diet change. Each dot represents a plasma sample derived from a single mouse. Data are represented as mean ± SEM, One-way ANOVA with Sidak’s post hoc test, **p < 0.01. **I** Evolution of weight over the time. Foxn1nu/nu Athymic-Nude mice bearing HT29-PHGDH-low derived tumors were treated as in (**F**) and mice weight was monitored each two days until experiment endpoint. Data are reported as relative to the average weight at the day of diet change and represented as mean ± SEM of at least three mice.

To further elucidate the relevance of exogenous Ser availability in the relationship with cancer cachexia and muscle atrophy, we evaluated the effect of reducing Ser plasma levels in in vivo CRC mice models. We adopted a restrictive dietary approach to modulate Ser and Gly plasma levels in Foxn1nu/nu Athymic-Nude mice previously injected with low-PHGDH HT29 cells (Fig. 2A). As already described [34, 38], feeding mice with a Ser/Gly-free diet (*-S-G* *diet*) for 10 days was sufficient to reduce Ser and Gly plasma levels (Fig. 2B). Of note, the *-S-G* dietary approach exacerbated tumor-related weight loss (Fig. 2C) and produced a specific reduction in skeletal muscle mass as measured by the quantification of gastrocnemius muscle volume (Fig. 2D, E) and the diameter of gastrocnemius muscle fibers (Fig. 2F, G). Interestingly, cross-sectional histological staining of muscle fiber showed that muscles from mice fed with *-S-G diet* display wider spaces between muscle fibers (Fig. 2F) and thinner fibers (Fig. 2G) compared to those in the control group (+*S*+*G diet*). To confirm the role of Ser in the observed wasting phenomenon, we evaluated the effect of adding Ser to the drinking water five days after diet change in *-S-G* *diet* fed mice (Fig. 2A). Interestingly, supplying Ser to HT29-PHGDH low-tumor-bearing mice brought Ser plasma up to normal levels (Fig. 2H) and countered the wasting phenomenon (Fig. 2I). To take into account the impact of the diminution of Ser plasma levels on the immune compartment during tumor progression, we assessed the effect of dietary Ser/Gly withdrawal in immunocompetent BALB/c mice previously injected with a syngeneic CRC-derived mouse cell line (CT26). The *-S-G* *diet* similarly reduced Ser plasma levels (Suppl. Figure 3A) and mice weight (Suppl. Figure 3B). Collectively, these data suggest that reduced Ser plasma availability affects skeletal muscle mass during tumor growth in different in vivo models.

### Tumor-muscle competition for serine availability occurs during cancer progression

**Fig. 3:**
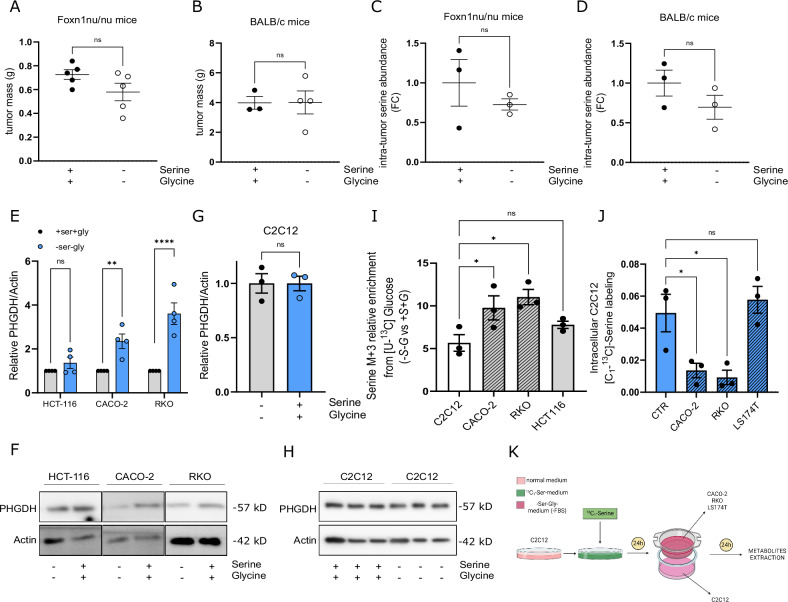
Tumor and muscle tissues compete for exogenous Ser availability during cancer progression. **A**, **B** Total tumor mass. Mass quantification of tumors obtained from HT29 low-PHGDH (**A**) and CT26 (**B**) tumor-bearing mice treated as in Fig. 2. Mass values were calculated based on tumor weight measured at the endpoint of the experiment. Each dot represents a plasma sample derived from a single mouse. Data are represented as mean ± SEM, Student’s t test. **C**, **D** Intra-tumor Ser levels. Relative Ser levels quantification in tumor tissue-derived samples from HT29 low-PHGDH (**C**) and CT26 (**D**) tumor-bearing mice treated as in Fig. 2. Metabolites from tissue samples were extracted as described in the “Materials and methods” section and tissue Ser content was quantified by GC-MS analysis. Each dot represents a plasma sample derived from a single mouse. Data are represented as mean ± SEM, Student’s t test. **E** PHGDH protein levels in CRC cell lines under Ser starvation. HCT-116, CACO2, and RKO cells were incubated in a medium containing (+Ser +Gly) or lacking (-Ser -Gly) Ser and Gly for 24 h before protein extraction. Cell lysates were analyzed by western blotting with the anti-PHGDH antibody. An anti-actin antibody was used to ensure equal protein loading. Data are expressed as relative to +Ser+Gly condition. Student’s t-test (*n* = 3). Each dot represents a single experiment. **F** Representative images of western blotting analysis evaluating PHGDH expression in HCT-116, CACO2, and RKO cells incubated in +Ser +Gly or -Ser -Gly medium for 24 h. **G** PHGDH protein levels in C2C12 myotubes under Ser starvation. C2C12 myotubes were incubated in a medium containing (+Ser +Gly) or lacking (-Ser -Gly) Ser and Gly for 6, 12, and 24 h before protein extraction. Cell lysates were analyzed by western blotting with the anti-PHGDH antibody. An anti-actin antibody was used to ensure equal protein loading. Data are expressed as relative to +Ser+Gly condition. Student’s t-test (*n* = 3). Each dot represents a single experiment. **H** Representative images of western blotting analysis evaluating PHGDH expression in C2C12 myotubes incubated in +Ser +Gly or -Ser -Gly medium for 24 h. **I** Ser synthesis pathway (SSP) activity analysis by [U-^**13**^C]-glucose labeling assay. Relative incorporation of [U-^13^C]-glucose-derived carbons in Ser. C2C12, CACO2, RKO, and HCT116 cells were incubated in a (-Ser -Gly) medium containing [U-^13^C]-Glucose for 3 h. Metabolite abundance and labeling enrichment were evaluated by GC-MS analysis. One-way ANOVA with Dunnett post hoc test (*n* = 3). Each dot represents a single experiment. **J** Competition for Ser availability between tumor and muscle cells. C2C12 myotubes were pre-incubated with a medium containing ^13^C_1_-Ser for 24 h before replacing growth medium with standard serum-starved-DMEM and adding tumor cells in the upper compartment of a Boyden chamber (pores diameter 0.4 µm). The fate of C2C12-derived ^13^C_1_-Ser was assessed by GC-MS analysis 24 h after tumor cell’s introduction. **K** Workflow for the ^13^C_1_-Ser labeling experiment described in **J**. One-way ANOVA with Tukey’s post hoc test (*n* = 3). Each dot represents a single experiment. Data are represented as mean ± SEM of n independent experiments, ns, not significant. *p < 0.05, **p < 0.01, ***p < 0.001.

Considering that Ser supports crucial functions in both healthy and tumor tissues [39], we investigated the effects of Ser/Gly dietary withdrawal on tumor growth. Interestingly, alongside the reported effects on total body weight, we did not observe any tumor mass reduction in in vivo models under Ser dietary restriction (Fig. 3A, B). By quantifying intra-tumor Ser content, we did not detect significant differences between tissues from mice fed with *+S* + *G* or *-S-G* diet (Fig. 3C, D). We, therefore, investigated modulations in PHGDH expression as a potential mechanism to compensate for extracellular Ser deprivation in tumors. By incubating tumor cells with a Ser/Gly-free medium, we observed increased PHGDH protein levels likely as an adaptative response to Ser starvation (Fig. 3E, F). Interestingly, low-PHGDH tumor cells CACO2 and RKO underwent a more pronounced increase in PHGDH levels under Ser/Gly withdrawal (Fig. 3E, F). Conversely, no increase in PHGDH levels was observed in C2C12 myotubes under Ser/Gly depletion (Fig. 3G, H), indicating muscle fibers do not compensate for exogenous Ser deprivation by enhancing SSP. We thus evaluated SSP activity in cells grown in the presence/absence of Ser and Gly for 48 h by performing a [U-^13^C]-glucose labeling experiment. The quantification of the M + 3 fraction of Ser following 3 h of incubation with [U-^13^C]-glucose indicated an increase in SSP activity in tumor cells as a consequence of Ser/Gly withdrawal that is more pronounced than muscle cells (Fig. 3I; Suppl. Figure 3C–F). This evidence led us to hypothesize that a metabolic competition may take place between tumor and muscle cells in conditions of exogenous Ser shortage. To test this hypothesis, we established an in vitro tumor-muscle cells co-culture system and followed the metabolic fate of C2C12-derived Ser after 24 h co-incubation. Briefly, we pre-incubated C2C12 myotubes with labeled [C_1_-^13^C]-Ser for 24 h to enrich muscle cells of labeled Ser. We then washed out labeled medium from C2C12 and introduced tumor cells (CACO2, RKO, LS174) to the system by placing them on the upper layer of a transwell insert (0.4 μm) upon C2C12 coltured cells (Fig. 3K). We monitored the metabolic fate of C2C12-derived Ser by extracting metabolites from the two cellular compartments after 24 h of co-incubation and analyzing samples by GC-MS. Interestingly, we measured lower intracellular [C_1_-^13^C]-Ser labeling in C2C12 myotubes co-incubated with low-PHGDH tumor cells (CACO2 and RKO) than in those incubated with high-PHGDH tumor cells (LS174) (Fig. 3J). Together, low-PHGDH expression determines exogenous Ser dependency in cancer. The consequent drop in Ser extracellular levels during tumor progression differentially affects tumor and muscle tissues (i.e., it greatly reduces muscle mass without restraining tumor growth).

### Ser starvation induces myotubes diameter reduction by inactivating the AKT/mTORC1 pathway

**Fig. 4:**
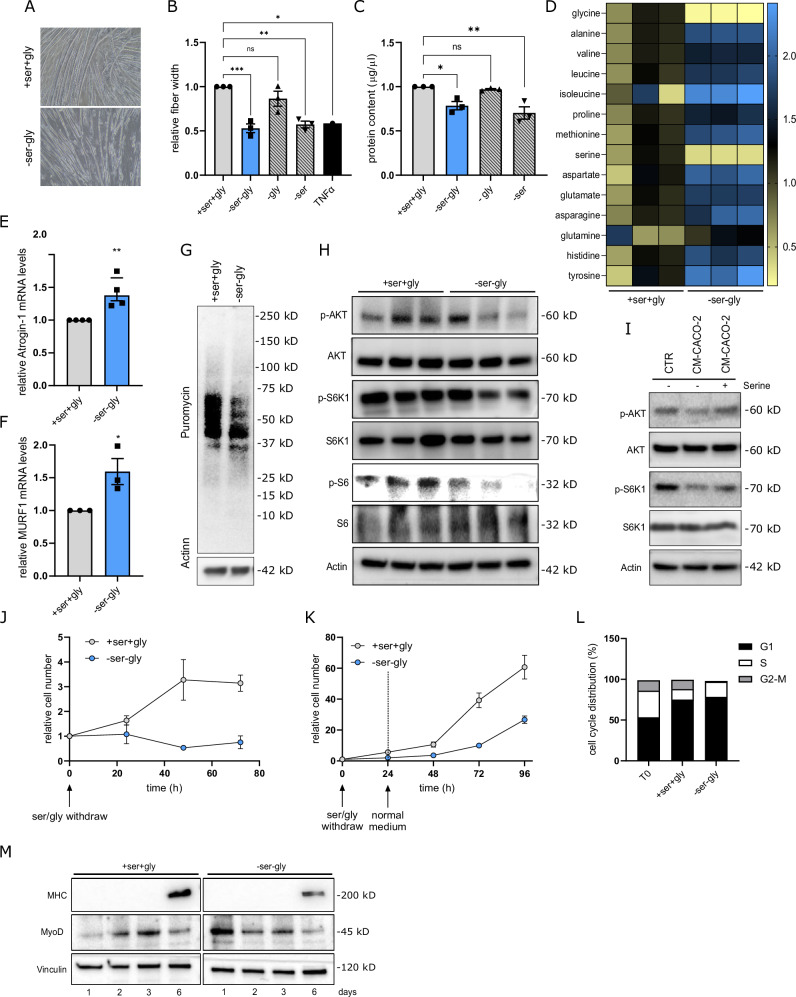
Limiting Ser availability in muscle fibers inactivates Akt/mTORC pathway leading to decreased protein synthesis and increased protein degradation. **A** Representative microscopic pictures of C2C12 myotubes. C2C12 myotubes were incubated in a medium containing (+Ser +Gly) or lacking (-Ser -Gly) Ser and Gly. Pictures were taken following 96 h of incubation. The image is representative of three independent experiments. **B** Alterations in myotubes width following Ser/Gly starvation. Relative C2C12 myotubes fibers width following 96 h of incubation with a medium containing (+Ser +Gly) or lacking (-Ser -Gly) Ser and/or Gly. 10 nM TNFα was used as wasting-inducer positive control. Data are expressed as relative to width values of myotubes grown in normal serum-free DMEM. One-way ANOVA with Tukey’s post hoc test (*n* = 3). Each dot represents a single experiment. **C** Alterations in total protein content in myotubes width following Ser/Gly starvation. C2C12 myotubes fibers were incubated for 96 h of incubation with a medium containing (+Ser +Gly) or lacking (-Ser -Gly) Ser and/or Gly before lysing cells for protein quantification analysis. Data are expressed as relative to protein content of myotubes grown in normal serum-free DMEM. One-way ANOVA with Tukey’s post hoc test (*n* = 3). Each dot represents a single experiment. **D** Myotubes AAs content following Ser/Gly starvation. C2C12 myotubes were incubated for 48 h with a medium containing (+Ser +Gly) or lacking (-Ser -Gly) Ser and/or Gly before metabolomic analysis. Metabolites were extracted as described in the “Materials and methods” section and intracellular AAs content was quantified by GC-MS. Data are expressed as relative to AAs levels measured in samples from cells incubated in serum-free DMEM. Data are reported as average of three independent experiments. **E**, **F** Atrogin-1 and MURF-1 expression levels. mRNA levels of Atrogin-1 (**E**) and MuRF-1 (**F**) were analyzed by quantitative RT-PCR in C2C12 myotubes following 48 h of incubation with +Ser +Gly/-Ser -Gly media. +Ser +Gly condition was used as comparator. Student’s t-test (*n* > 3). Each dot represents a single experiment. **G** Total protein synthesis in C2C12 myotubes under Ser/Gly starvation. Protein synthesis was evaluated on the incorporation of puromycin into newly synthesized proteins. C2C12 myotubes were incubated with +Ser +Gly/-Ser -Gly media for 24 h, starved in Hank’s balanced salt solution (HBSS) for 1 h, and subsequently reactivated (30 min) in a medium containing puromycin (10 mg/mL). Cell lysates were analyzed by western blotting with the anti-puromycin antibody. An anti-actin antibody was used to ensure equal protein loading. The image is representative of three independent experiments. **H** Phosphorylation of AKT and mTORC1 targets S6 and S6K1 in C2C12 myotubes under Ser/Gly starvation. C2C12 myotubes were incubated with +Ser +Gly/-Ser -Gly media for 6, 16, and 24 h before analyzing cell lysates by western blotting. An anti-actin antibody was used to ensure equal protein loading. The image is representative of three independent experiments. **I** Phosphorylation of AKT and S6K1 in C2C12 myotubes incubated in low Ser containing CM. C2C12 myotubes were incubated with boiled CM derived from CACO2 cells supplemented or not with exogenous Ser (0.4 mM) for 24 h before analyzing cell lysates by western blotting. An anti-actin antibody was used to ensure equal protein loading. The image is representative of three independent experiments. **J** Proliferation curves of C2C12 cells cultured in presence or absence of Ser/Gly. Proliferation curves of C2C12 cell lines grown in medium with Ser/Gly (+ser+gly, gray dots) or without Serine/Gly (-ser-gly, blue dots). Cell number was assessed every 24 h (*n* = 3). **K** Proliferation curves of C2C12 cells following 24 h of Ser/Gly starvation. C2C12 cells were incubated for 24 h in presence (+ser+gly, gray dots) or absence (-ser-gly, blue dots) of Ser/Gly before assessing cell proliferation in normal medium by counting cell number every 24 h (*n* = 3). **L** Cell Cycle distribution of C2C12 cells cultured in presence or absence of Ser/Gly. Quantitative analysis of relative cell number in G1, S, and G2/M of C2C12 cells incubated in grown in medium with or without Serine/Gly for 24. C2C12 cells were stained with Propidium Iodide (PI) to analyze the cell cycle distribution by flow cytometry. Quantitation was determined by measuring the area corresponding to the indicated phase. **M** Time-dependent changes in MHC and MYOD protein levels during differentiation under Ser/Gly starvation. C2C12 myotubes were incubated with differentiation media (dialyzed FBS 2%) in presence or not of Ser/Gly for 1, 2, 3, 6 days before analyzing cell lysates by western blotting with the anti-MHC and anti-MYOD antibodies. An anti-Vinculin antibody was used to ensure equal protein loading. The image is representative of three independent experiments. Data are represented as mean ± SEM of *n* independent experiments, ns, not significant. *p < 0.05, **p < 0.01, ***p < 0.001.

To investigate the molecular consequences of Ser deprivation in muscle cells, we analyzed the effect of withdrawing extracellular Ser from the culture medium of C2C12-derived muscle fibers. To minimize possible confounding effects due to Gly-to-Ser conversion, we also removed Gly from the culture medium. By depriving C2C12 myotubes of Ser and Gly (*-Ser-Gly*) for 72 h, we observed a relevant decrease in fiber width compared to standard culture conditions (+*Ser+Gly*) (Fig. 4A, B). Interestingly, the sole Ser (*-Ser*) but not Gly (*-Gly*) withdrawal caused myotubes diameter reduction to a similar extent as *-Ser-Gly* conditioning (Fig. 4B), demonstrating the specific role of Ser in maintaining muscle integrity. Noteworthy, the reduction in myotubes diameter by Ser/Gly depletion was comparable to that observed following 48 h of treatment with the cachexia inducer factor TNF-α (Fig. 4B).

One of the most relevant features of cancer cachexia is the progressive protein loss in skeletal muscle as a result of both increased protein degradation [40] and decreased protein synthesis [41]. Coherently, incubating C2C12 myotubes with *-Ser-Gly* medium, as well as *-Ser* medium, significantly reduced total intracellular protein content (Fig. 4C) and resulted in the accumulation of intracellular free non-Ser/Gly AAs (Fig. 4D), suggesting the activation of protein degradation systems. Atrogin-1 and MuRF-1 are described as chief regulators of ubiquitin-driven protein breakdown in skeletal muscle during wasting [42]. In accordance, we observed an increase in the expression of Atrogin-1 (Fig. 4E) and MuRF-1 (Fig. 4F) in C2C12 myotubes under Ser/Gly starvation. Concurrently, the incubation with *-Ser-Gly* medium also reduced protein translation in C2C12 myotubes as evaluated by puromycin incorporation assay [43] (Fig. 4G).

Modulations in the AKT/mTORC1 axis in muscle fibers distresses cellular homeostasis by dysregulating autophagy and affecting protein synthesis/protein degradation balance [44]. In particular, mTORC1 activation is strictly regulated by nutrients availability [45]. In agreement, we observed lower activating phosphorylation levels of AKT and the mTORC1 substrates S6 and S6K1 in C2C12 cells under Ser/Gly starvation than in standard culture conditions (Fig. 4H). To assess the contribution of tumor cells to the identified molecular mechanism we incubated C2C12 myotubes for 72 h with CM and assessed the activation of the AKT/mTORC1 pathway. In keeping with previous evidence, C2C12 myotubes incubated with CM from low-PHGDH cells CACO-2 exhibited lower AKT and S6K1 phosphorylation than control conditions (Fig. 4I). Importantly, the sole Ser supplementation to the CACO-2 CM was sufficient to rescue AKT and S6K1 phosphorylation in C2C12 myotubes (Fig. 4I). Together, these data indicate that low-Ser conditions create a depleted environment that is sensed by muscle myotubes through the lack of activation of the AKT/mTORC1 pathway, resulting in reduced protein synthesis and ultimately affecting muscle mass.

### Ser starvation affects C2C12 growth dynamics and impairs the ability of C2C12 myoblasts to differentiate into myotubes

In addition to increased protein degradation and decreased protein synthesis, muscle regeneration is impaired in cancer cachexia [46, 47]. Myoblasts undergoing myogenic differentiation must first exit from the cell cycle and switch from a proliferative to a differentiative state [48]. By investigating cell proliferation of progenitor myoblasts under Ser limitation, we observed that removing Ser and Gly from the culture medium strongly impairs C2C12 cell proliferation (Fig. 4K). Interestingly, incubating C2C12 fibers in a *-Ser-Gly* medium for a short time (24 h) is sufficient to definitively affect C2C12 proliferation (Fig. 4L), indicating that limiting the availability of these two amino acids commits myoblasts to a non-proliferative state. Coherently, the analysis of cell cycle distribution of C2C12 myoblasts indicates that culturing cells in a Ser/Gly-deprived medium blocks their entry into the mitotic phase, inducing an abnormal accumulation of cells in the S phase (Fig. 4M). Importantly, the process of terminal myogenic differentiation requires the cell cycle arrest in G0 to be coupled with increased expression of specific transcription factors regulating differentiation, including MyoD. In accordance with the impaired proliferative potency myoblasts under Ser/Gly limitation, incubating C2C12 cells in a *-Ser-Gly* medium for 24 h induces an initial strong increase in MyoD protein levels (Fig. 4N). As myocyte progenitors differentiate, they progressively express late-skeletal muscle differentiation markers and myogenic factors such as myosin heavy chain (MHC) [49]. To better characterize how Ser/Gly deprivation affects the regeneration ability of muscle, we investigated the time-dependent expression dynamic of early (myogenic differentiation 1 (MyoD)) and late (myosin heavy chain (MHC)) skeletal muscle differentiation markers in C2C12 cells incubated in the presence or not of Ser and Gly during differentiation. Interestingly, despite the higher levels of MyoD in the early phases of differentiation, myoblasts cultured under Ser/Gly starvation fail to appropriately differentiate as indicated by lower levels of MHC after 6 days (Fig. 4N). This data suggests that the absence of extracellular Ser/Gly induces a cellular stress that commits myoblasts toward myogenic differentiation, but it fails to finalize muscle differentiation, resulting in impaired regeneration of atrophied muscles.

### Ser-Gly starvation affects mitochondrial metabolism and causes oxidative stress in muscle fibers

**Fig. 5:**
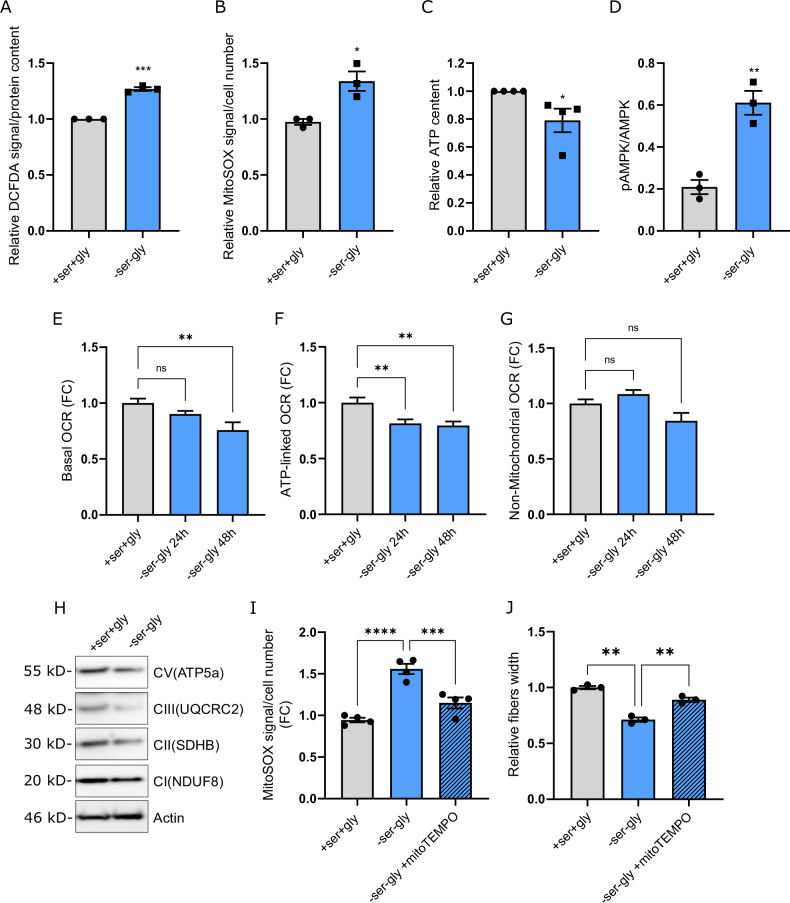
Ser-Gly starvation impairs mitochondrial metabolism and causes oxidative stress in muscle fibers. **A** Total ROS levels in C2C12 myotubes under Ser/Gly starvation. Intracellular ROS were quantified in C2C12 myotubes incubated with +Ser +Gly/-Ser -Gly media for 24 h by staining with DCFDA probe and analyzing them by FACS. Data are expressed as relative to +Ser+Gly condition. Student’s t-test (*n* = 3). Each dot represents a single experiment. **B** Mitochondrial ROS levels in C2C12 myotubes under Ser/Gly starvation. Mitochondrial ROS were quantified in C2C12 myotubes incubated with +Ser +Gly/-Ser -Gly media for 24 h by staining with MitoSOX probe and analyzing by FACS. Data are expressed as relative to +Ser+Gly condition. Student’s t-test (*n* = 3). Each dot represents a single experiment. **C** Intracellular ATP levels in C2C12 myotubes under Ser/Gly starvation. C2C12 myotubes were incubated with +Ser +Gly/-Ser -Gly media for 24 h before analyzing ATP content. Data are expressed as relative to +Ser+Gly condition. Student’s t-test (*n* = 3). Each dot represents a single experiment. **D** Phosphorylation of AMPK in C2C12 myotubes under Ser/Gly starvation. C2C12 myotubes were incubated with +Ser +Gly/-Ser -Gly media 24 h before analyzing cell lysates by western blotting with the anti-phospho-AMPK and anti-AMPK antibody. AMPK phosphorylation levels are reported as relative to total AMPK levels. An anti-actin antibody was used to ensure equal protein loading. Student’s t-test (*n* = 3). Each dot represents a single experiment. **E**–**G** Oxidative metabolism in myotubes under Ser/Gly starvation. Oxygen consumption rate (OCR) was measured in C2C12 myotubes previously incubated with +Ser +Gly/-Ser -Gly media for 24 and 48 h. **E** Basal OCR was calculated as the OCR values before Oligomycin injection. **F** ATP-linked respiration was calculated by subtracting OCR values obtained following Oligomycin injection to basal OCR. **G** Non-mitochondrial respiration was calculated as the OCR values after Rotenone/Antimycin A injection. Data are normalized to those from C2C12 cells incubated with +Ser +Gly medium. One-way ANOVA with Dunnett’s post hoc test (*n* = 3). Each dot represents a single experiment. **H** Mitochondrial respiration complexes in C2C12 myotubes under Ser/Gly starvation. C2C12 myotubes were incubated with +Ser +Gly/-Ser -Gly media for 24 h before analyzing cell lysates by western blotting with the anti-OXPHOS complexes antibody. An anti-actin antibody was used to ensure equal protein loading. The image is representative of three independent experiments. **I** Mitochondrial ROS accumulation in Ser starved C2C12 myotubes upon mitoTEMPO treatment. C2C12 cells were incubated with +Ser +Gly/-Ser -Gly media 24 h in presence or not of 2.5 µM mitoTEMPO before quantifying mitochondrial ROS by staining them with MitoSOX probe and analyzing by FACS. Data are normalized to those from C2C12 cells incubated with +Ser +Gly medium. Two-way ANOVA with Tukey’s post hoc test (*n* = 3). Each dot represents a single experiment. **J** Quantification of Ser starved C2C12 myotubes upon MitoTEMPO treatment. C2C12 cells were incubated with +Ser +Gly/-Ser -Gly media 72 h in presence or not of 2.5 µM mitoTEMPO before quantifying fiber width. Data are normalized to those from C2C12 cells incubated with +Ser +Gly medium. Two-way ANOVA with Tukey’s post hoc test. Data are represented as mean ± SEM of *n* independent experiments, ns, not significant. *p < 0.05, **p < 0.01, ***p < 0.001.

Cancer cachexia is generally associated with an alteration in redox homeostasis, mitochondrial dysfunction, and loss of oxidative capacity in skeletal muscle [50], finally leading to reactive oxygen species (ROS) accumulation and increased protein oxidation [51, 52]. Considering the importance of the Ser/Gly metabolism in maintaining intracellular redox homeostasis [53–55], we hypothesized that lower free Ser availability occurring during cancer progression may promote muscle wasting also by increasing the oxidative stress in myotubes. Accordingly, by incubating C2C12 fibers in a -*Ser-Gly* medium for 48 h, we measured a significant increase in both total (Fig. 5A) and mitochondrial ROS (Fig. 5B). This indicates that Ser/Gly starvation affects redox homeostasis in muscle cells and suggests an impairment in mitochondrial functionality. Coherently, Ser starvation also resulted in decreased intracellular ATP content (Fig. 5C) and a concomitant increase in AMPK phosphorylation (Fig. 5D). Assessing mitochondrial activity by Seahorse analysis on C2C12 fibers previously incubated with -*Ser-Gly* conditions for 24 h and 48 h, we observed that concomitant Ser/Gly starvation decreases basal oxygen consumption rate (OCR) (Fig. 5E) and ATP-linked respiration (Fig. 5F), while non-mitochondrial OCR was unaffected (Fig. 5G). In agreement, by western blot analysis, we observed a reduction in CV, CIII, and CII mitochondrial complexes in C2C12 myotubes following Ser/Gly starvation (Fig. 5H). To confirm the role of oxidative stress in myotubes diameter reduction under Ser/Gly starvation, we investigated the effect of scavenging mitochondrial ROS in C2C12 myotubes. Remarkably, treating C2C12 with the mitochondria-targeted antioxidant mitoTEMPO decreased mitochondrial ROS content (Fig. 5I) and rescued physiological myotubes width under -*Ser-Gly* culture conditions (Fig. 5J). In summary, these results demonstrate that limited environmental Ser affects redox homeostasis and mitochondrial functionality in myotubes potentially contributing to muscle wasting during cancer cachexia.

## Discussion

Tumor-derived signals governing the anabolic/catabolic balance in peripheral tissues play an important role in cancer cachexia [33]. In particular, increasing evidence underlined the existence of an “auto-cannibalism strategy” according to which the stimulation of a negative energy balance in peripheral tissues results in the release of nutrients in the bloodstream, which in turn is functional to support tumor growth [2]. Different tumor-derived factors have been recognized to carry out this role in cancer cachexia. For example, in CRC, the release of the High Mobility Group Box 1 protein (HMGB1) from tumor cells affects host energy metabolism and induces muscle autophagy. Interestingly, the temporal release of HMGB1 in the serum during cancer progression is associated with increased muscle autophagy with a consequent alteration in plasma-free amino acids and increased circulating glutamine availability to be used by cancer cells as an energy source [56]. Along with tumor-derived signals, cancer cells’ high consumption of nutrients and energy substrates may establish a competition for diminishing resources between tumors and other organs, further exacerbating tissue wasting in cachectic patients [57]. Indeed, tumor cells strongly rely on exogenous supply of essential and non-essential AA to provide adequate cellular building blocks, energy, and reducing agents supporting proliferation [58, 59]. This intensive consumption result in the depletion of specific nutrients in the whole organism. Specifically, alterations in free AA plasma profile have been described in CRC [11, 60, 61].

Among others, cancer cells are particularly greedy for Ser [16], which is required to sustain proliferative metabolism by supporting nucleotide and macromolecule biosynthesis, maintaining cellular redox balance and modulating the epigenetic landscape of cancer cells [62]. Besides, Ser and Gly are also strictly necessary to preserve skeletal muscle integrity and functionality under stress conditions [21]. These AAs are essential to repair damaged myofibers and maintain their homeostasis after injury [22]. Different studies reported a significant reduction of Ser plasma levels in cachectic CRC-bearing mice [25, 26], skeletal muscle protection from cancer-induced wasting following Gly administration [23]. We thus investigated the establishment of a metabolic competition for Ser availability between highly proliferative tumor cells and muscle myotubes. In this scenario, decreased Ser plasma levels due to cancer cell consumption emerged to impact muscle physiology finally resulting in enhanced muscle wasting and cancer cachexia.

Starting from bioinformatic analysis in GEO from mice with tumor-induced cachexia, we identified a significative dysregulation in metabolic-related genes expression in skeletal muscle. In particular, alterations in mitochondrial and amino acids metabolism specifically characterized the cachectic group. Considering the detrimental role of Ser availability in supporting CRC progression, we focused on Ser for subsequent analysis. By exploiting in vitro and in vivo models of cancer-induced muscle wasting, we found that Ser availability strongly affects muscle trophism. In vitro Ser deprivation, either cancer-induced or obtained by manipulating media composition, induces myotubes diameter reduction. In vivo, feeding mice with a Ser/Gly-free diet worsens tumor-induced weakness. Ser and Gly are interchangeable AAs; however, our in vitro data demonstrate specific atrophic effects under Ser, but not Gly, media depletion. This evidence aligns with in vivo results showing that the sole Ser supplementation in drinking water of mice fed with a Ser/Gly-free diet is sufficient to revert weight loss. By investigating the molecular basis underpinning this atrophic response under Ser starvation, we uncovered that incubating skeletal muscle cells in a Ser/Gly-free medium disrupts protein homeostasis by impairing the AKT/mTOR signaling pathway. The AKT/mTOR pathway is a nutrient-sensing system that responds to intracellular levels of different AAs, including Ser [63, 64]. This adaptive response results in the inhibition of protein synthesis and the accumulation of free AAs in myofibers and is known to be impaired during cancer cachexia [44, 65].

Numerous studies have demonstrated that mitochondrial dysregulation plays a crucial role in muscle wasting in the context of cancer cachexia. In particular, a balanced Ser metabolism is crucial in preserving oxidative stress balance [23] and ensures an adaptive mitohormetic response in skeletal muscle [21]. In accordance, we observed that limiting Ser availability in myotubes in vitro affects the antioxidant properties of skeletal muscles, impairing mitochondrial functionality and increasing ROS. Treating muscle fibers with the mitochondria-targeted antioxidant mitoTEMPO attenuates myotubes diameter reduction under Ser/Gly-deprived conditions. Together these data indicate that impaired protein homeostasis and altered antioxidant balance contribute to myotubes diameter reduction under environmental Ser limitation.

Along with the suppression of protein synthesis pathways and enhanced/accelerated protein breakdown, impaired muscle regeneration is equally involved in the wasting state associated with cancer cachexia [66]. In contrast to what is observed in healthy individuals where muscle injury activates skeletal muscle stem cells to differentiate, promoting muscle regeneration, cancer cachexia is associated with the loss of myogenic precursors and impaired muscle regenerative potential [67]. Different tumor-derived cytokines such as NF-κB signaling activating factors (i.e., tumor necrosis factor (TNF)) [46], CXCL1 [68], and inflammatory stimuli enhancing CCAAT/Enhancer Binding Protein beta (C/EBPβ) expression [69] have been described to antagonize muscle regeneration and interfere with muscle satellite cell homeostasis during cancer cachexia. While previous studies reported that skeletal muscle cells are able to sense nutrient availability [70] and that nutrient limitation affects myoblast differentiation, the role of nutrient availability in governing myogenic differentiation during cancer cachexia is still poorly defined. By investigating the effect of Ser limitation in myoblast differentiation, we observed an early arrest of cell proliferation coupled with the induction of MyoD expression in myoblast. However, this initial cell commitment to the myogenic lineage induced by Ser starvation is insufficient to finalize a successful terminal myogenic differentiation. These results align with previous studies reporting that MyoD prepares myoblasts for efficient myogenic differentiation, but is not directly involved in supporting terminal myoblast proliferation [71]. Tumor-dependent environmental Ser limitation could, therefore, induce damage-like stimuli triggering initial myoblast differentiation that, however, is not effective in finalizing muscle regeneration of atrophied cachectic muscles.

In conclusion, this study describes a new metabolic competition for limited Ser availability occurring in CRC between tumor and skeletal muscle cells. Environmental Ser limitation during CRC progression affects protein homeostasis and redox balance in muscle tissue, resulting in myotubes diameter reduction, impaired myogenic differentiation, and muscle wasting. Interestingly, the CRC-cachectic potential is strongly related to PHGDH expression in tumor cells and exogenous Ser dependency, opening new possibilities for prognostic approaches. These data could help to design new nutritional interventions as a novel means to manage the cachectic condition in CRC patients. Indeed, based on the PHGDH status of primary tumors, patients could be turned to a specific nutritional approach to minimize the complications related to cachexia.

## Materials and methods

### Transcriptomic analysis of muscle fibers from murine cachectic individuals CRC-dependent

We performed a survey of Gene Expression Omnibus (GEO, https://www.ncbi.nlm.nih.gov/geo) to interrogate transcriptomic data of skeletal muscle from mice in which cachexia was induced by injection of C26 CRC cell lines. RNA expression datasets from adipose tissues of cachectic individuals associated with other pathological conditions were discarded.

Two murine microarray datasets, GSE24112 (Illumina MouseWG-6 v2.0 expression beadchip, https://www.ncbi.nlm.nih.gov/geo/query/acc.cgi?acc=GSE24112), and GSE48363 (Affymetrix Mouse Gene 1.0 ST Array, https://www.ncbi.nlm.nih.gov/geo/query/acc.cgi?acc=GSE48363) were selected, downloaded and the expression matrices statistically analyzed. Each probe ID was associated to the gene name which belongs to. For GSE24112, identification of Differentially Expressed Genes (DEGs) was accomplished by comparing 45281 probes from 4 normal samples, extracted from the quadriceps muscle of female CD2F1 mice, with those of 4 affected by “severe weight loss” (15%) and colon cancer induced by C26 cell lines injection. The available 4 “early weight loss” (10%) samples were not included in the analysis. For GSE48363, DEGs were identified by comparing 21212 probes from three pooled total RNA samples of healthy control muscles with 3 pooled total RNAs from muscles of CT26 tumor-bearing mice. To identify DEGs, the Mann–Whitney U-Test, *p* < 0.05 and Fold Change ≤ −1.3 or ≥1.3 statistical criteria were applied for each dataset. Pathway analysis of DEGs was performed on www.metascape.org as described by Zhou et al. [72]. DEGs were compared to those included in the metascape gene-sets, as GO processes, KEGG pathways, Reactome, MSigDB, etc, to define their involvement in specific biological processes. Gene sets whose members were significantly overrepresented were reported as biological discoveries, while those that did not meet the minimal statistical requirements were removed. Hypergeometric test and Benjiamini–Hochberg *p*-value correction were applied for ontology purposes.

### Cell models

Colorectal carcinoma cells HCT-116, HT29, HCT8, CACO-2, LS174T, RKO, and mouse colorectal carcinoma cells CT26 were obtained from ATCC and cultured in high glucose (4.5 g/L) Dulbecco’s modified Eagle’s medium (DMEM) (Merck Sigma #D5671) supplemented with 10% fetal bovine Serum (EuroClone #ECS0180L), 1% penicillin and streptomycin (EuroClone #ECB3001D), and 2 mM L-glutamine (Merck Sigma #G7513). Mouse C2C12 myoblasts were obtained from ATCC and cultured in DMEM with 10% fetal bovine serum (FBS). Once reached full confluency, cell differentiation was induced by switching to 2% horse Serum (HS) for 5 days. Cells were routinely grown in DMEM in a humidified atmosphere with 5% CO_2_ at 37 °C. All cell lines were confirmed to be mycoplasma-free by the Mycoalert detection kit (Lonza).

### Mouse models

All animal experiments were approved by the Italian ethical committee of Animal Welfare Office of Italian Work Ministry and conformed to the legal mandates and Italian guidelines for the care and maintenance of laboratory animals (authorization number: 311/2022-PR). Mice were randomized before cell inoculation; all samples were analyzed blinded. Sample size was determined using G power software in compliance with the 3R system: Replacement, Reduction, Refinement. For syngeneic experimental model, CT26 cells were implanted by bilateral subcutaneous injections (1 × 10^5^ cells per flank in 100 µl PBS) into 6 three-week-old BALB/c male mice (Charles River, UK). For experiments with immunodeficient mice, HT29R cells were inoculated by bilateral subcutaneous injections (1 × 10^6^ cells per flank in 100 µl PBS) into 6 three-week-old Foxn1nu/nuAthymic nude mice (Envigo RMS srl, USA). Mice were maintained on standard diet (Mucedola srl) and water ad libitum and monitored daily until palpable tumor nodules were present. Two weeks after tumor cells injection, tumor-bearing animals were placed on experimental conditions. Mice were weighed each two days and average tumor volume was calculated three times/week using the formula: ${{\rm{volume}}}={\frac{{{\rm{length}}\; \times\; {\rm{width}}}}{2}}^{2}$. Tumor-bearing mice were fed with a control or Ser/Gly-free diet (50 g/mouse/week). The experimental diets applied were provided from TestDiet (Richmond, IN, USA) and were formulated as previously described as “Diet 1-Control” and “Diet 1-SG-free” in [19]. Briefly, the control diet contains all the essential amino acids, including Ser, Gly, glutamine, arginine, cystine, and tyrosine. The Ser/Gly-free diet has the same formulation of the control diet but lacks Ser and Gly, compensated by a proportional addition of the other amino acids to reach an equal amount of amino acid content.

### In vivo ultrasound imaging

Ultrasound (US) imaging was carried out at Laboratory of Genetic Engineering for the production of Animal Models (LIGeMA) at the Animal Facility of the University of Florence. US imaging was performed to evaluate gastrocnemius muscle volume with Vevo LAZR-X (Fujifilm Visualsonics) platform. Axial 3D scans of the tumors were performed in B-Mode by using a 55-MHz transducer. During the procedure mice were anesthetized by isoflurane (2%) and placed on a heated pad at 37 °C in prone position. Respiration rate, ECG, and body temperature were monitored during the procedure. Tumor volumes were analyzed by using Vevo Lab software (Fujifilm Visualsonics). The volumes were measured delineating the ROI (Region Of Interest) for every axial slide using Vevo LAB.

### Hematoxylin-Eosin staining (H&E staining)

Bilateral gastrocnemius muscle was integrally harvested, and three pieces of the gastrocnemius muscle in each group were randomly selected for subsequent Hematoxylin-Eosin (H&E) staining. Gastrocnemius muscles were collected, fixed in 4% neutral-buffered formalin for 24 h, processed and embedded in paraffin (FFPE). Sections of 7 µm were cut to perform H&E staining with Leica ST5010 Autostainer XL. Images were obtained with a slide scanner (Aperio LV1, Leica Biosystems) and analyzed with ImageScope Software. Skeletal muscle fiber area was measured automatically by ImageJ software.

### Conditioned media from cancer cells

CRC cancer cells were grown to 70–80% confluence and incubated for 48 h with serum-free medium to obtain the corresponding conditioned media (CM), which were subjected to boiling at 95 °C for 20 min, then filtered and used fresh or stored at 20 °C for further analysis.

### In vitro fiber width quantification

Myotubes were incubated with cancer cells-derived CM or experimental media for 96 h before taking pictures of three representative areas/well with phase contrast microscopy at ×10 magnification. Myotube width was measured using the ImageJ imaging system.

Experimental media were formulated starting from MEM medium (Gibco #21090) supplemented with 10% dialyzed FBS, 1% MEM vitamins (Merck Sigma #M6895), 2 mM L-glutamine (Merck Sigma), D-glucose (Merck Sigma #G8644) to reach a final concentration 17 mM, 1% penicillin and streptomycin (EuroClone #ECB3001D), and 2 mM L-glutamine (Merck Sigma #G7513). To generate “+Ser +Gly” media, 0.4 mM Ser (Merck Sigma #S5386) and 0.4 mM Gly (Merck Sigma #G8790) were added; “-Ser -Gly” media lack these two amino acids.

### Cell cycle analysis

The cell cycle analysis was carried out using cytofluorimetric method. Briefly, C2C12 cells were grown in the presence or absence Ser/Gly for 24 h, then cells were fixed in 70% cold ethanol. After cells were resuspended in a buffer containing 0.05 mg/mL propidium iodide (PI), 5 μg/mL RNAase A, 0.2% v/v Nonidet P-40, 0.1% sodium citrate. Samples were analyzed by FACScan Flow Cytometer Apparatus (BD Biosciences, San Jose, CA) and ModFit Software (BD Biosciences) was used to determine the cell cycle distribution.

### Protein extraction, quantification, and Western blot analysis

Cell lysates were prepared in RIPA lysis buffer (50 mM Tris-HCl pH 7.5, 150 mM NaCl, 100 mM NaF, 2 mM EGTA, 1% Triton X-100) supplemented with protease inhibitors cocktail (Merck Sigma #P8340) and phosphatase inhibitor cocktail (Merck Sigma #P5726), and subsequently centrifuged at 14,000 rpm and 4 °C for 10 min. Frozen tissue samples were fragmented and resuspended in RIPA lysis buffer. Protein concentration was quantified by bicinchoninic acid (BCA) assay (Merck Sigma #BCA1).

For western bot analysis, 15–30 μg of total proteins was then loaded on SDS-PAGE gels and transferred to PVDF membranes (BioRad #1704157). Membranes were incubated for 1 h at room temperature in blocking buffer (5% non-fat dry milk (SantaCruz Biotechnology #sc-2325) in PBS-Tween 0.1%), and incubated at 4 °C over-night with primary antibodies (against either PHGDH (Thermo Fisher Scientific #PA5-54360), PSAT1 (Thermo Fisher Scientific # PA522124), PSPH (Thermo Fisher Scientific # PA596863), Phospho-Akt (Ser473) (Cell Signaling Technology #9271), AKT A (Cell Signaling Technology #9272), MHC (SantaCruz Biotechnology #sc376157), MyoD1 (Cell Signaling Technology #13812), Phospho-S6 Ribosomal Protein (Ser240/244) (Cell Signaling Technology # 5364), S6 Ribosomal Protein (Cell Signaling Technology # 2217), Phospho-p70-S6 Kinase (Thr389) (Cell Signaling Technology # 9205), pS6K1 p70-S6 kinase (Cell Signaling Technology # 9202), TRB-3 (SantaCruz Biotechnology #sc-365842), LC3 (GTX82986), AMPK (Cell Signaling Technology #2532), p-AMPK (Thr172) (Cell Signaling Technology #2535), OXPHOS complexes (total OXPHOS Rodent WB Antibody Cocktail (abcam, #ab110413), β-Tubulin (SantaCruz Biotecnology #sc-5286), β-Actin (Santa Cruz Biotechology #sc-477778). All primary antibodies were diluted 1:1000 in PBS-Tween 0.1% containing 5% bovine Serum albumin (BSA) (Merck Sigma #A7906)). The following day, after washing in PBS-Tween 0.1%, membranes were incubated for 1 h at room temperature with horSeradish peroxidase-conjugated anti-mouse (Santa Cruz Biotechology #sc-2005) or anti-rabbit (Santa Cruz Biotechology #sc-2357) (diluted 1:2500 in PBS-Tween 0.1% containing 1% BSA) antibodies. Bound antibodies were detected using Clarity Western ECL Substrate (BioRad #1705061) and images were acquired using Amersham Imager 600 luminometer (Amersham, Buckinghamshire, UK). Quantification of bands was carried out by using the Amersham quantification software. β-Actin or β-Tubulin were used as loading control of total protein lysates.

### Real-time PCR

Total RNA was purified using the RNeasy Plus Mini Kit (Qiagen #74134) according to manufacturer’s instructions. Total RNA was quantified at NanoDrop Microvolume Spectrophotometer and Fluorometer (Thermo Fisher Scientific). Strands of cDNA were synthesized from 1 µg of total extracted RNA using the QuantiTect cDNA Reverse Transcription Kit (Qiagen #205311) and the MJ Mini Personal Thermal Cycler (Bio-Rad), according to manufacturer’s instructions. Quantification of mRNA expression levels of specific targets was evaluated by real-time PCR (RT-PCR) using QuantiFast SYBR Green PCR kit (Qiagen #204054). Amplification reactions were run on CFX96TM Touch Real-Time PCR Detection System (Bio-Rad) according to the manufacturer’s instructions. Data were reported as relative quantity with respect to the reference sample using the 2-ΔΔCt method. β2 microglobulin (β2M) was used to normalize the data. The specific primers for mRNA analysis were provided from Thermo Fisher Scientific. The nucleotide sequences of the specific primers used are:

MURF1 ((forward) 5′-GGT GCC TAC TTG CTC CTT GT-3′, (reverse) 5′-CTG GTG GCT ATT CTC CTT GG-3′).

Atrogin1 ((forward) 5′-CAG CCT GCC TGT GTG CTT AC-3′, (reverse) 5′-CTT GCG AAT CTG CCT CTC TG-3′).

β2M ((forward) 5′-AGT ATG CCT GCC GTG TGA AC-3′, (reverse) 5′-GCG GCA TCT TCA AAC CTC CA-3′).

### Protein synthesis (SUnSET) assay

Puromycin incorporation into synthesized protein was quantified to assess protein synthesis as previously described [43]. Next, cells were plated in six-well plates in triplicate wells in the standard medium; 24 h before the analysis, cells were serum starved in +Ser +Gly or -Ser -Gly media. Subsequently, cells were washed with PBS and starved one additional hour in Hanks’ Balanced Salt Solution (HBSS). Next, cells were reactivated with a medium containing dialyzed FBS in the presence or absence of Ser/Gly. Throughout the last 10 min of reactivation, puromycin was added to the medium at a concentration of 10 μg/ml. Cell lysates were collected, and protein extracts were prepared in RIPA lysis buffer. Puromycin incorporation was evaluated by western blot analysis using an anti-puromycin antibody (Merck, MABE343).

### Ser uptake assay

Cells were plated in six-well plates in triplicate wells in standard medium 24 h before the analysis; duplicate plates were seeded for normalization by cell counting. Ser uptake was evaluated by incubating cells in a Serum-starved medium with Ser substituted for [1-2^14^C]-Ser for 15 min. Cells were subsequently washed with PBS and lysed with 0.1 mol/L NaOH. Cell lysates were transferred to a scintillation vial and measured on the scintillation counter (Tri-Carb 2800TR, PerkinElmer). The radioactive signals were normalized to cell number.

### Intracellular ATP quantification

ATP levels were determined using an ATP Detection kit-luminescence Assay Kit (CAY-700410-1-BioVision, Milpitas, CA) according to the manufacturer’s instructions. All data were normalized on cell protein content.

### Total ROS production

Cells were incubated with 10 μg/mL of 2′,7′-dichlorodihydrofluorescein diacetate (H2DCF-DA, Invitrogen, Thermo Fisher Scientific #D399) for 30 min at 37 °C. Cells were then lysed in 200 μL RIPA lysis buffer and centrifuged at 14,000 rpm and 4 °C for 10 min. 150 μL of cell lysates were transferred in a 96-well plate and fluorescence was measured at wavelength excitation/emission 485 nm/535 nm using the Biotek Synergy H1 microplate reader. Obtained results were normalized on total protein content quantified with the BCA assay.

### Mitochondrial ROS (mtROS) measurements

Cells were plated in six-well plates in triplicate wells in standard medium 48 h before the analysis; 24 h before mtROS analysis, cells were treated with mitoTEMPO 2.5 µM for 24 h. Cells were then stained with 2.5 µM MitoSOX (Invitrogen, Thermo Fisher Scientific #M36008) in Serum-free medium for 15 min at 37 °C. Cells were then trypsinized, resuspended in complete media, and washed twice with PBS, before collection. Fluorescence was analyzed at wavelength excitation/emission 510 nm/580 nm using BD FACSCanto™ II Flow Cytometry System (BD Bioscience, Franklin Lake, NJ, USA).

### GC-MS analysis

Cells were seeded in six-well plates in triplicate wells in standard medium 24 h before analysis. For isotopomer distribution assays, fresh media containing [C_1_-^13^C]-Ser (final concentration 0.4 mM) or [U-^13^C]-glucose (final concentration 17 mM) were formulated as follows and added to cells before metabolite extraction at different time points (30 min, 1 h, 3 h for [U-^13^C]-glucose experiments, and 3 h, 8 h, 24 h for [C_1_-^13^C]-Ser experiments). [C_1_-^13^C]-Ser labeled medium was formulated starting from MEM medium (Gibco #21090) supplemented with 10% dialyzed FBS, 1% MEM vitamins (Merck Sigma #M6895), 1% penicillin and streptomycin (EuroClone #ECB3001D), 2 mM L-glutamine (Merck Sigma #G7513), D-glucose (Merck Sigma #G8644) to reach a final concentration 17 mM, 0.4 mM Gly (Merck Sigma #G8790). [U-13C]-glucose labeled medium was formulated starting from no-glucose DMEM (ThermoFisher Scientific #11966025) supplemented with 10% dialyzed FBS, 1% penicillin and streptomycin, 2 mM L-glutamine. Metabolites from cells were extracted by quenching plates in liquid nitrogen to arrest metabolic activity, and lysing cells in 800 µl of a cold (−20 °C) solution of methanol 80% in water (containing 1 mg noravline/ml as internal standard). Metabolites from culture media were extracted by adding 100 µl of cold solution of methanol 100% (containing 0.5 mg noravline/ml as internal standard) to 100 µl of media previously centrifuged for 10 min at 1.200 rpm. Metabolites from plasma were extracted by adding 100 µl of cold solution of methanol 100% (containing 0.5 mg noravline/ml as internal standard) to 100 µl of of plasma. Plasma was isolated from blood samples quickly collected via cardiac puncture in heparin-containing tubes by centrifuging for 15 min at 2.500 rpm. Metabolites from tissues were extracted by resuspending mashed tissue fractions (50–70 mg) in 400 µl of cold solution of methanol 50% in water and 400 µl of chloroform and sonicating them three times (10 s each) in ice. Tissue for metabolites extraction were rapidly dissected, washed in ice-cold saline (NaCl 0.9%), frozen in liquid-nitrogen, and samples were stored at −80 °C until processing.

Samples were vortexed at for 10 min at 4 °C and then centrifuged at 4 °C and 14,000 rpm for 10 min. Polar metabolites were derivatized with 30 µl of a solution 20 µg/ml methoxyamine (Merck Sigma #226904) in pyridine (Merck Sigma #270970) for 90 min at 37 °C. Then, 45 µl of N-(tert-butyldimethylsilyl)-N-methyl-trifluoroacetamide, with 1% tert-butyldimethylchlorosilane (Merck Sigma #375934) were added in each sample and incubated for 60 min at 37 °C. GC-MS runs were with helium as carrier gas with a flow rate of 0.6 mL/min. The split inlet temperature was set to 250 °C and the injection volume of 1 μL. The GC oven temperature ramp was from 70 to 280 °C. The first temperature ramp was from 70 to 140 °C at 3 °C/min. The second temperature ramp was from 140 to 180 °C at 1 °C/min. Finally, the latest temperature ramp was from 180 to 280 °C at 3 °C/min. For the Quadrupole, an EI source (70 eV) was used. The ion source and transfer line temperatures were set, respectively, to 250 and 290 °C.

For the determination of relative metabolite abundances, the integrated signal of all ions for each metabolite fragment was normalized by the signal from norvaline and sample protein content isolated by resuspending the protein layer in 50 µl NaOH 200 mM, vertexing for 15 min at 96 °C, and centrifuging at 4 °C 14,000 rpm for 15 min. Protein abundance was measured by BCA assay. For labeling experiments, the measured distributions of mass isotopomers were corrected for natural abundance of ^13^C using IsoCor software [73] and the abundance of each isotopologue is indicated as normalized to the sum of all possible isotopologues [74].

### Seahorse analysis

Cells were seeded in XFe96 cell culture plates (6–8 technical replicates per condition) in 80 μL of standard medium and let to adhere at 37 °C. Standard medium was then replaced with “+Ser +Gly” or “-Ser-Gly” (see “In vitro fiber width quantification” section) for the following 24–48 h. 1 h before the analysis, media were replaced with “+Ser +Gly” or “-Ser-Gly” with adjusted PH at 7.4. Cells were incubated for 1 hour at 37 °C in atmospheric CO_2_ conditions to pre-equilibrate cells. OCR and ECAR analysis were performed using Seahorse XF Cell Mito Stress Test (Agilent # 103015-100) according to manufacturer’s instructions. Mitochondrial drugs were utilized as follows: 0.8 μM of oligomycin, 1 μM of FCCP, 1 μM of rotenone, and 1 μM of antimycin A were injected three times subsequently at the times indicated. Results were normalized to protein content. Basal respiration is calculated as the average rate measurement before injection minus the average of non-mitochondrial respiration rate. ATP-linked respiration is calculated as the average of the basal respiration minus OCR measurement after Oligomycin injection.

### Quantification and statistical analysis

Statistical data analysis was performed with GraphPad Prism version 9.0 (GraphPad Software) on at least three biological replicates for each experiment (see figure legends for details). Statistical analysis was performed assuming Gaussian distribution of residuals. Data are indicated as mean ± SEM, as indicated in the figure legends. Mathematical outliers were detected using Grubb’s test (alpha = 0.1) and identified values were removed.

All methods were performed in accordance with the relevant guidelines and regulations.

## Supplementary information

Supplemetal Figure Legend

Supplemental Fiure 1

Supplemental Figure 2

Supplemental Figure 3

Original Wester Blot

## Data Availability

Data sharing is not applicable to this article as no datasets were generated or analyzed during the current study. All data generated or analyzed during this study are included in this published article or available from the corresponding author on reasonable request.
