# Supplementary material for: Limiting serine availability during tumor progression promotes muscle wasting in cancer cachexia
Source: Cell Death Discov. 2024 Dec 21;10:510. doi: 10.1038/s41420-024-02271-1 (PMC11662032; doi:10.1038/s41420-024-02271-1)
Supplement: Supplementary file 1 — Supplemetal Figure Legend [file 41420_2024_2271_MOESM1_ESM.docx]

**Suppl. Figure 1:**

**Pathway analysis of Differentially Expressed Genes (DEGs) from two murine datasets, GSE24112 and GSE48363**

**Suppl. Figure 2:**

**(A) Exogenous serine uptake in CRC cell lines**. [1-2^14^C]-serine incorporated by CRC cells expressed in counts per minute (CPM) and normalized on cell number. Data are expressed as relative to HCT-8 cell line levels. One-way ANOVA with Tukey’s post hoc test (n = 3). **(B) Gly media content** analyzed by GC-MS on media collected following 48h of incubation with cells. Data are expressed as relative to serum-free DMEM. One-way ANOVA Tukey’s post hoc test (n = 3). Each dot represents a single experiment. **(C) Gly media content-myotubes width correlation analysis.** Pearson correlation between Gly content in CM from CRC cell lines and C2C12 myotubes width following 96h of incubation with CM. Data are normalized on values from C2C12 myotubes incubated in normal serum-free DMEM. **(D) PHGDH expression in HT29-high and HT29-low cells.** mRNA levels of PHGDH in HT29-high and HT29-low cells analyzed by quantitative RT-PCR using parental HT29-high cells as comparator. Student’s t test (n=3). Each dot represents a single experiment. **(E) Exogenous serine uptake in HT29-high and HT29-low clones.** [1-2^14^C]-serine incorporated by HT29-high and HT29-low cells expressed in counts per minute (CPM) and normalized on cell number. Student’s t test (n=3). Each dot represents a single experiment.

**Suppl. Figure 3:**

**(A) Relative plasma Ser levels.** Ser levels were quantified by GS-MS analysis in plasma from Balb/c mice bearing CT26-derived tumors and fed with +S+G or -S-G diet. Each dot represents a plasma sample derived from a single mouse. Data are represented as mean ± SEM, Student’s t test, **p<0.01. **(B) Relative mice weight.** Balb/c mice bearing CT26-derived tumors were fed with a +S+G or -S-G since tumor was palpable and mice weight was assessed each two days until experiment endpoint. Weight values are normalized on +S+G diet condition. Each dot represents a single mouse. Data are represented as mean ± SEM, Student’s t test, **p<0.01. **(C-F) Incorporation of a [U-^13^C]-glucose carbons in Ser** in C2C12 (C), CACO-2 (D), RKO (E), HCT-116 (F) cells. Muscle fibers and tumor cells were incubated in a medium containing [U-^13^C]-glucose for 3 h before assessing intracellular metabolite labeling enrichment (n = 3). **(G) Schematic representation of C2C12-tumor cells co-culture experiment setting.** C2C12 myotubes were pre-incubated with labeled [C_1_-^13^C]-Ser for 24 h. Tumor cells were then platedon the upper layer of a transwell insert (0.4um) upon C2C12. C2C12-derived labeled carbons were analyzed after 24h of incubation by GC-MS.
