# Supplementary figures and images for: Limiting serine availability during tumor progression promotes muscle wasting in cancer cachexia
Source: Cell Death Discov. 2024 Dec 21;10:510. doi: 10.1038/s41420-024-02271-1 (PMC11662032; doi:10.1038/s41420-024-02271-1)

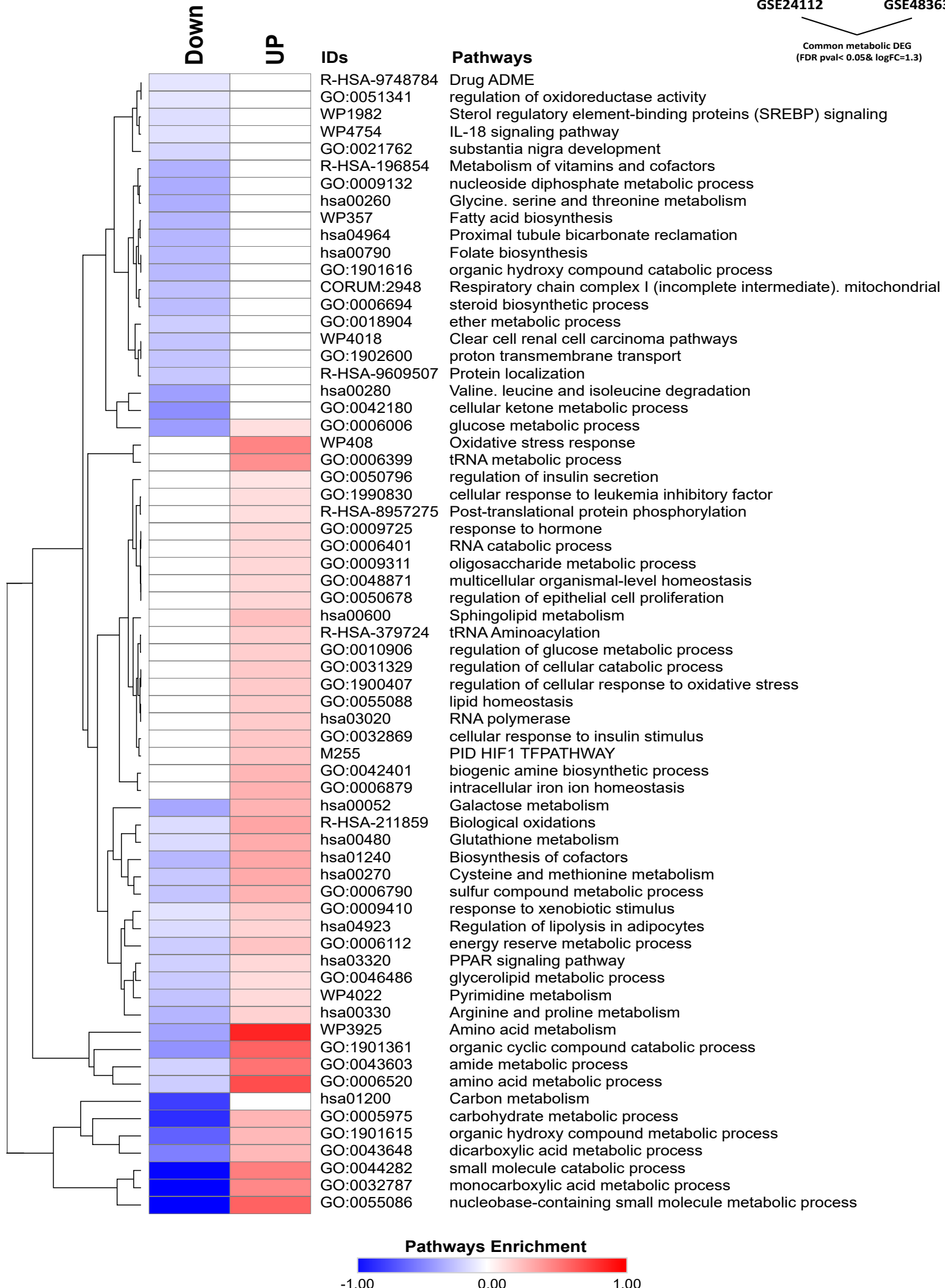

Supplement: Supplementary file 2 — Supplemental Fiure 1 [file 41420_2024_2271_MOESM2_ESM.pdf]

A

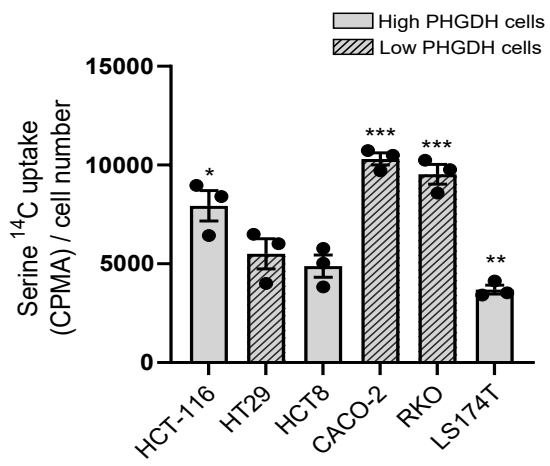

B

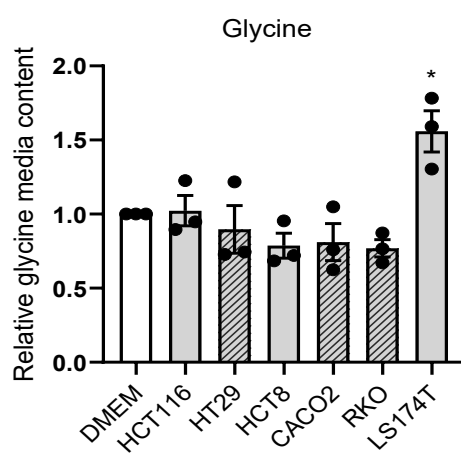

C

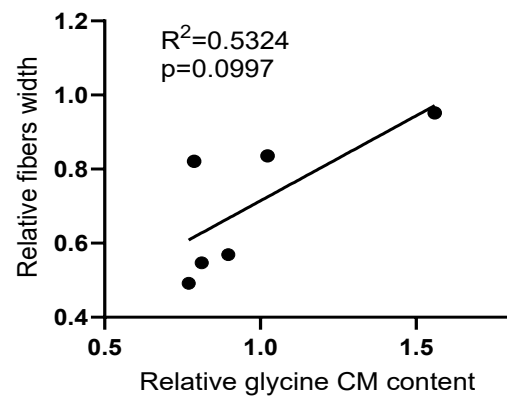

D

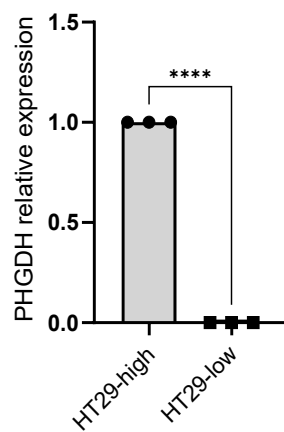

E

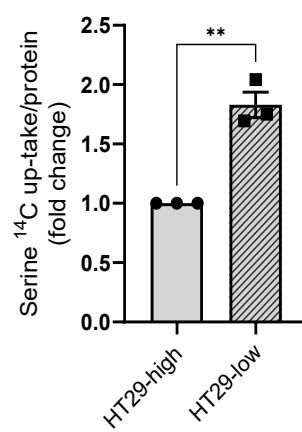

Supplement: Supplementary file 3 — Supplemental Figure 2 [file 41420_2024_2271_MOESM3_ESM.pdf]

A

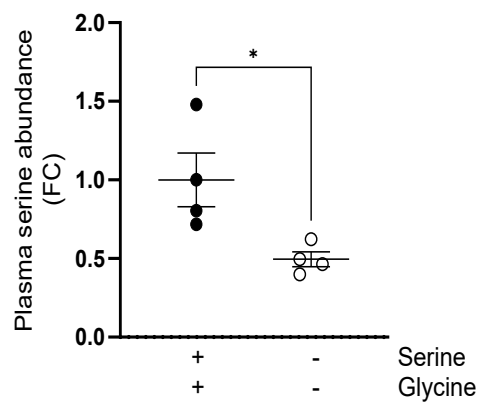

B

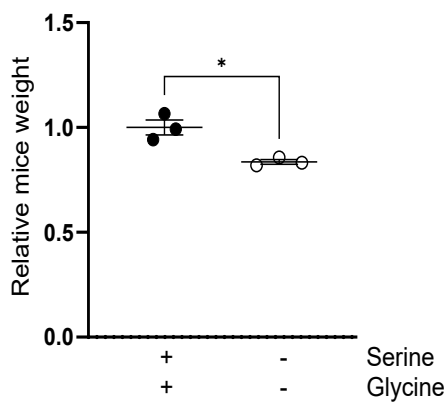

C

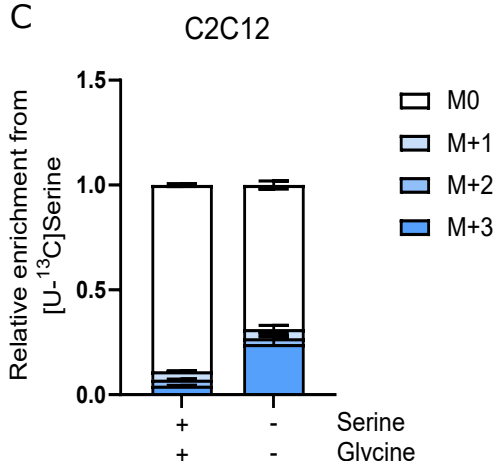

D

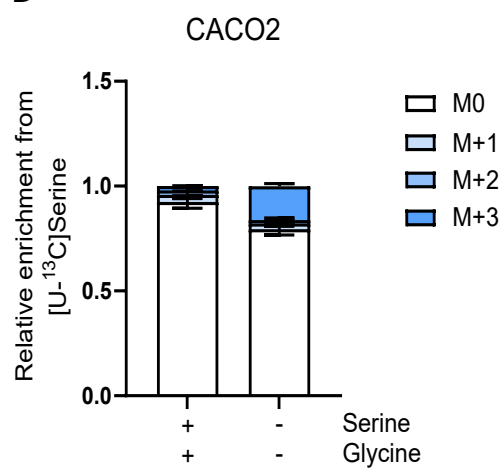

E

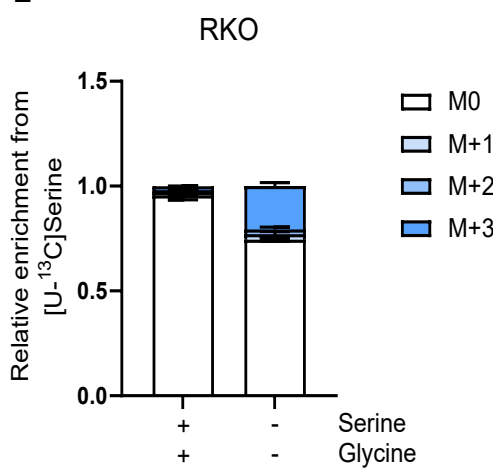

F

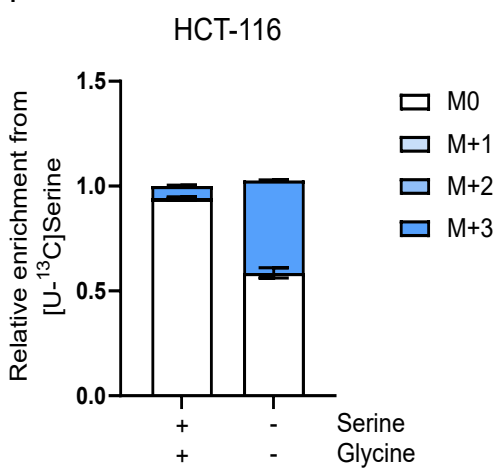

Supplement: Supplementary file 4 — Supplemental Figure 3 [file 41420_2024_2271_MOESM4_ESM.pdf]
