# Supplementary material for: Limiting serine availability during tumor progression promotes muscle wasting in cancer cachexia
Source: Cell Death Discov. 2024 Dec 21;10:510. doi: 10.1038/s41420-024-02271-1 (PMC11662032; doi:10.1038/s41420-024-02271-1)
Supplement: Supplementary file 5 — Original Wester Blot [file 41420_2024_2271_MOESM5_ESM.pdf]

Fig. 1B

Precision Plus Protein™ All Blue
Prestained Protein Standards, BioRad

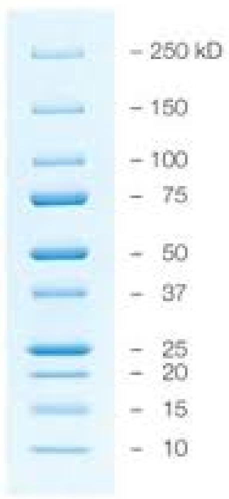

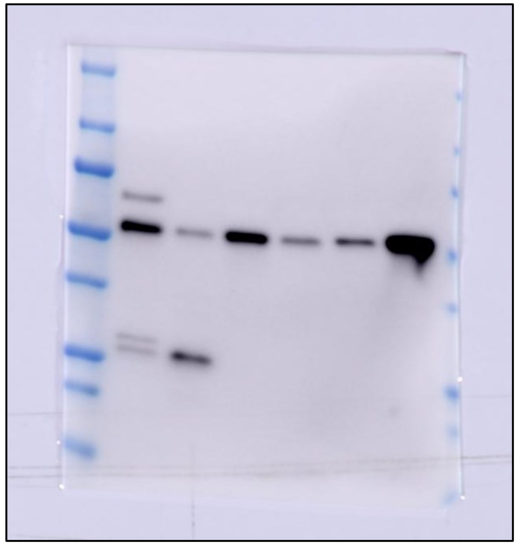

Anti-PHGDH
(MW: 57)

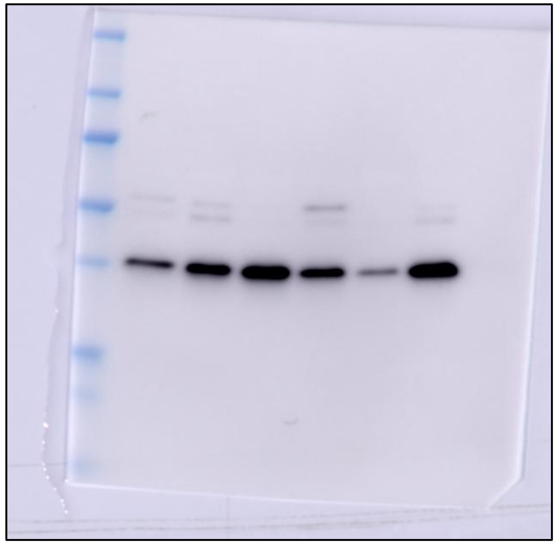

Anti-PSAT1
(MW: 40)

Anti-Actin
(MW: 40)

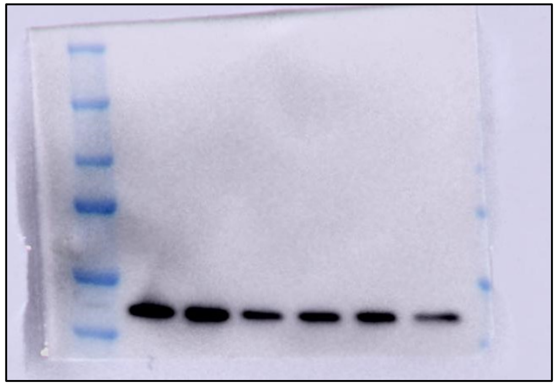

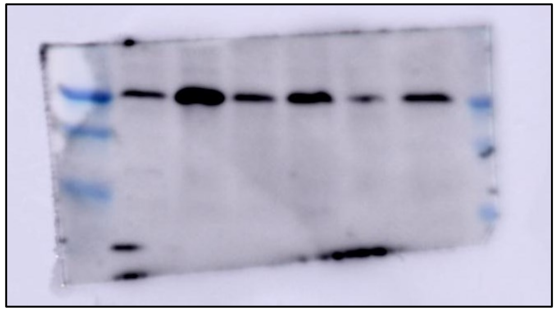

Anti-PSPH
(MW: 25)

Fig. 3F

Precision Plus Protein™ All Blue
Prestained Protein Standards, BioRad

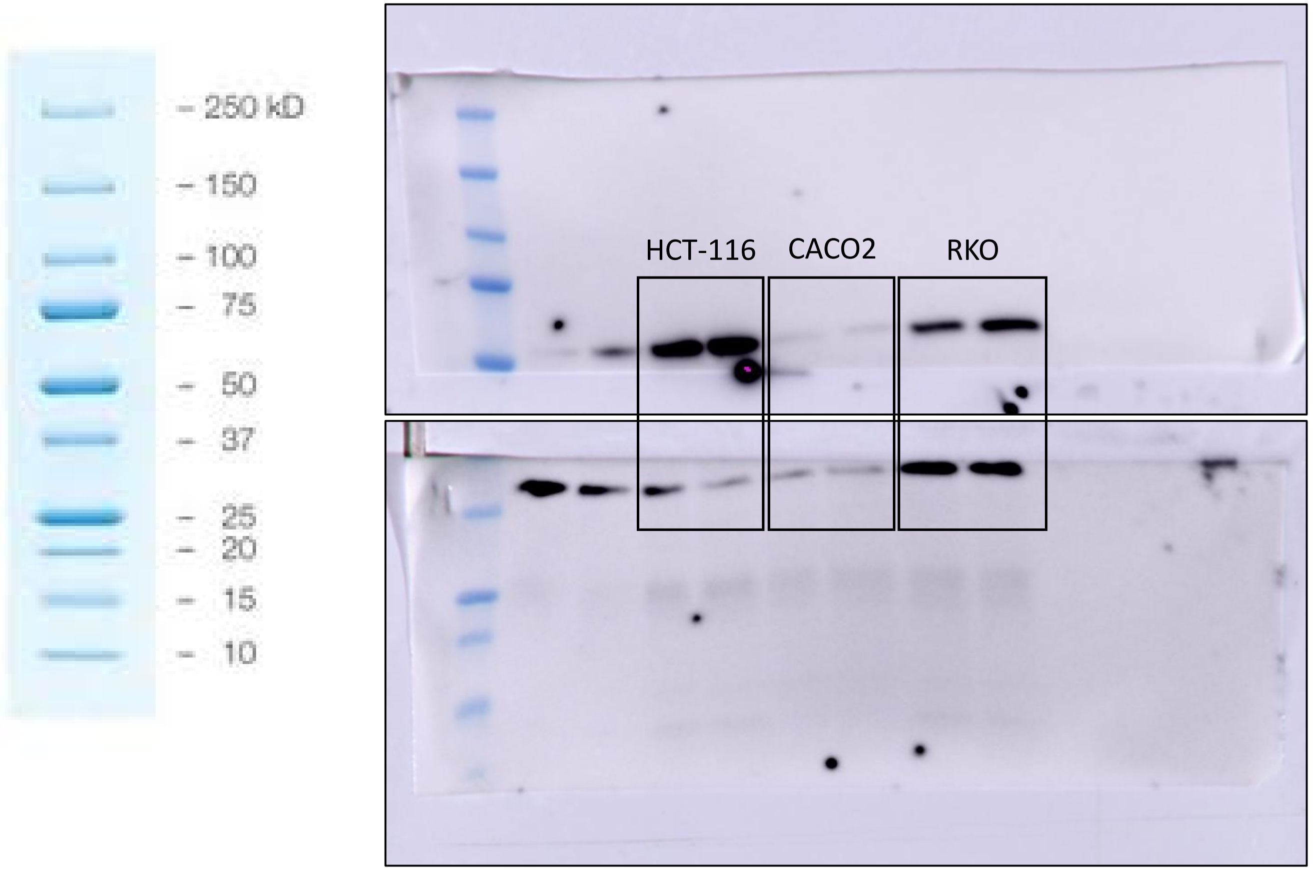

Anti-PHGDH
(MW: 57)

Anti-Actin
(MW: 40)

Fig. 3H

Precision Plus Protein™ All Blue
Prestained Protein Standards, BioRad

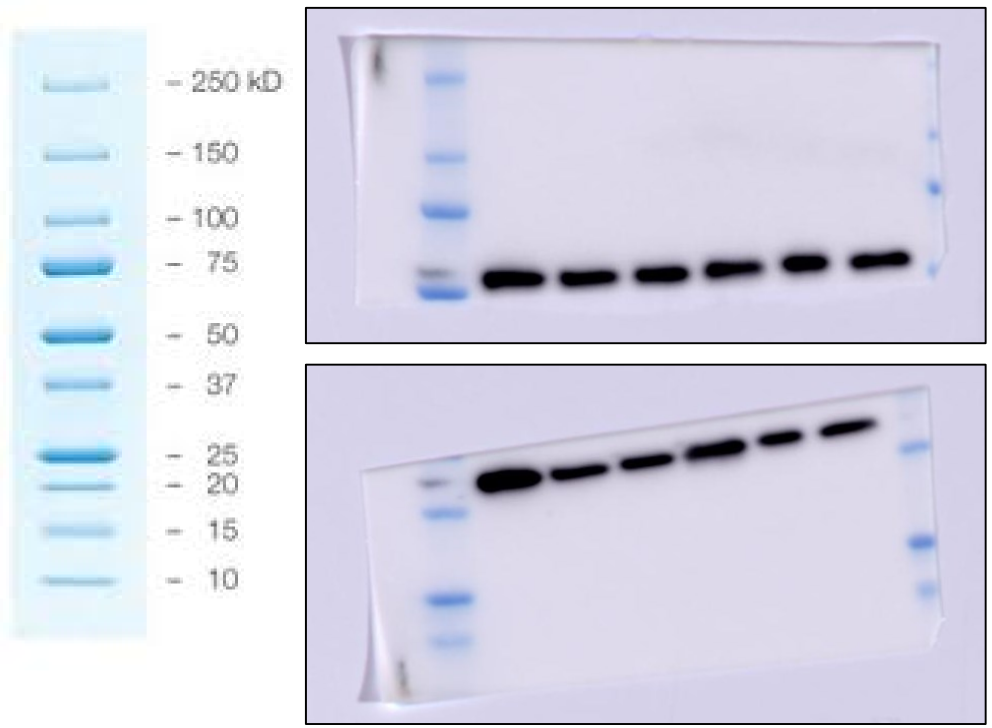

Anti-PHGDH
(MW: 57)

Anti-Actin
(MW: 40)

Fig. 4G

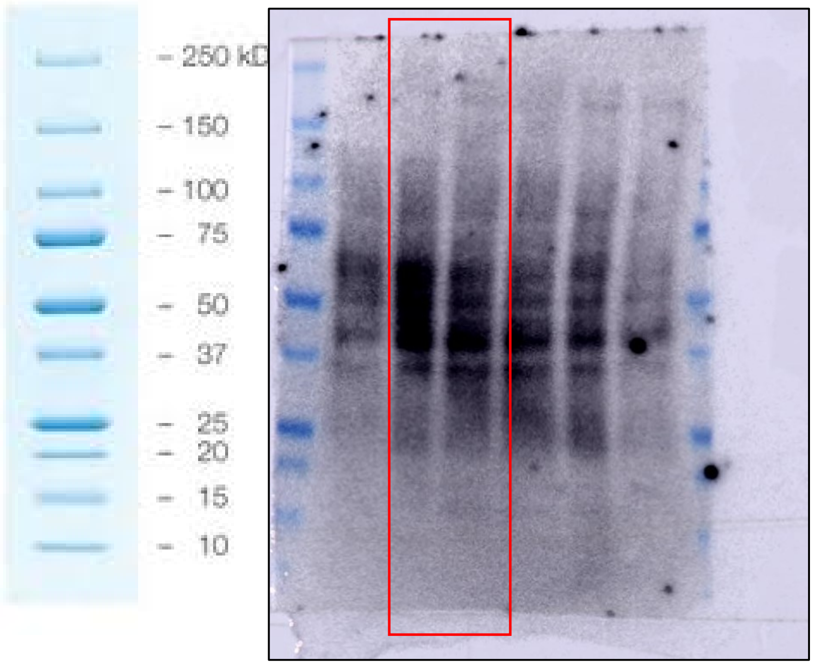

Anti-puromycin (MW: 60)

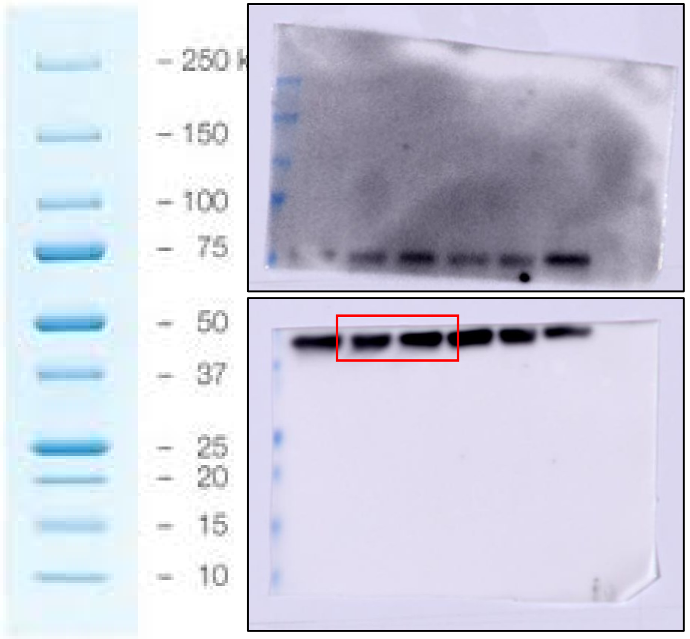

Anti-Tubulin (MW: 50) not shown in the paper

Anti-Actin (MW: 40)

Fig. 4H

Precision Plus Protein™ Dual Color Standards

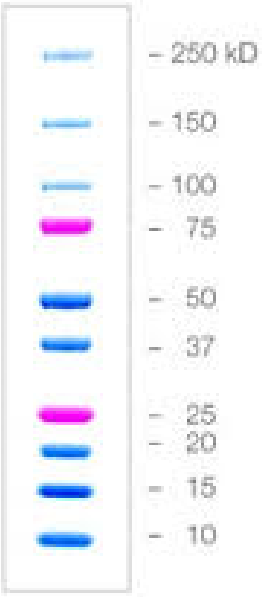

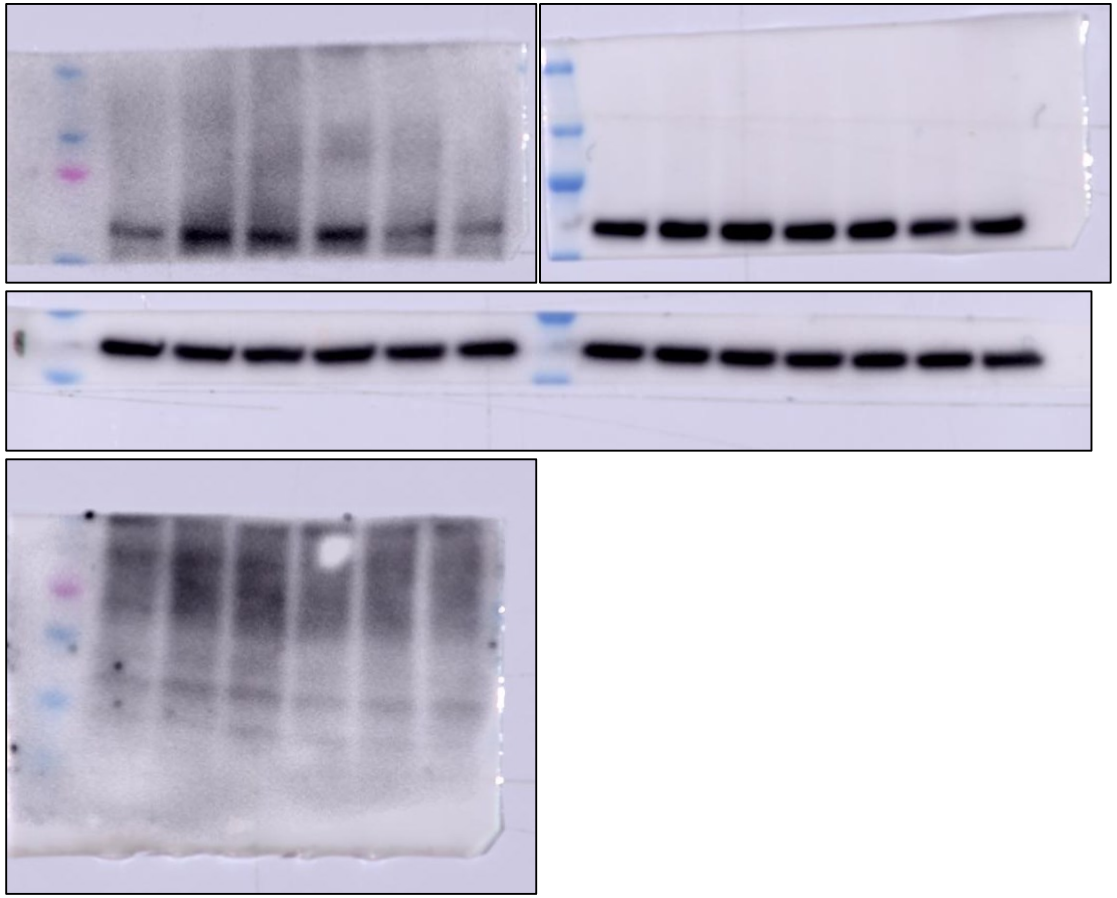

Anti-phosphoAKT (MW: 60)

Anti-AKT (MW: 60)

Anti-Actin (MW: 40)

Anti-S6 (MW: 32) not shown in the paper

Fig. 4H

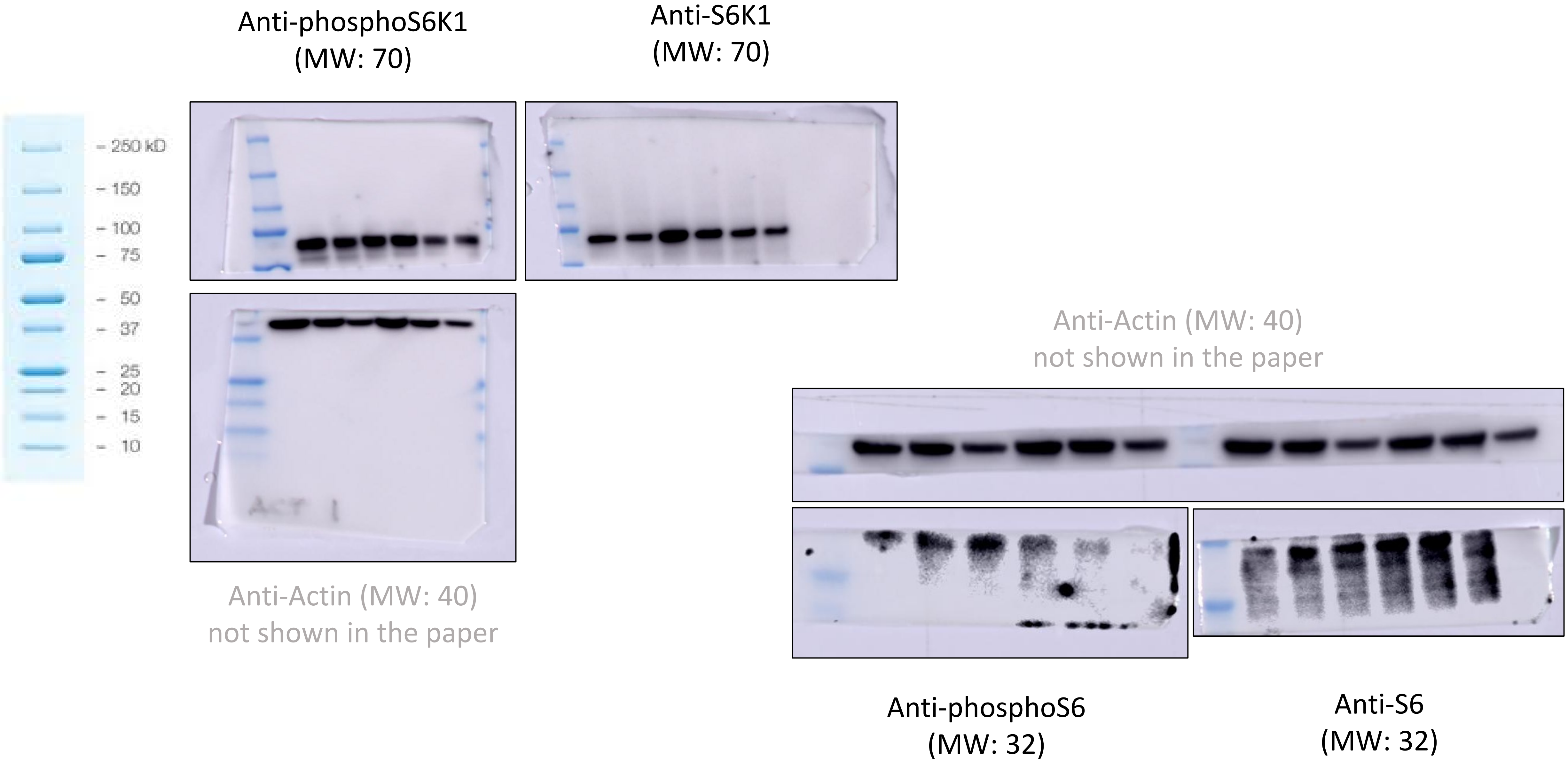

Anti-phosphoS6K1
(MW: 70)
Anti-S6K1
(MW: 70)
250 kD
150
100
75
50
37
25
20
15
10
Anti-Actin (MW: 40)
not shown in the paper
Anti-Actin (MW: 40)
not shown in the paper
Anti-phosphoS6
(MW: 32)
Anti-S6
(MW: 32)

Fig. 4I

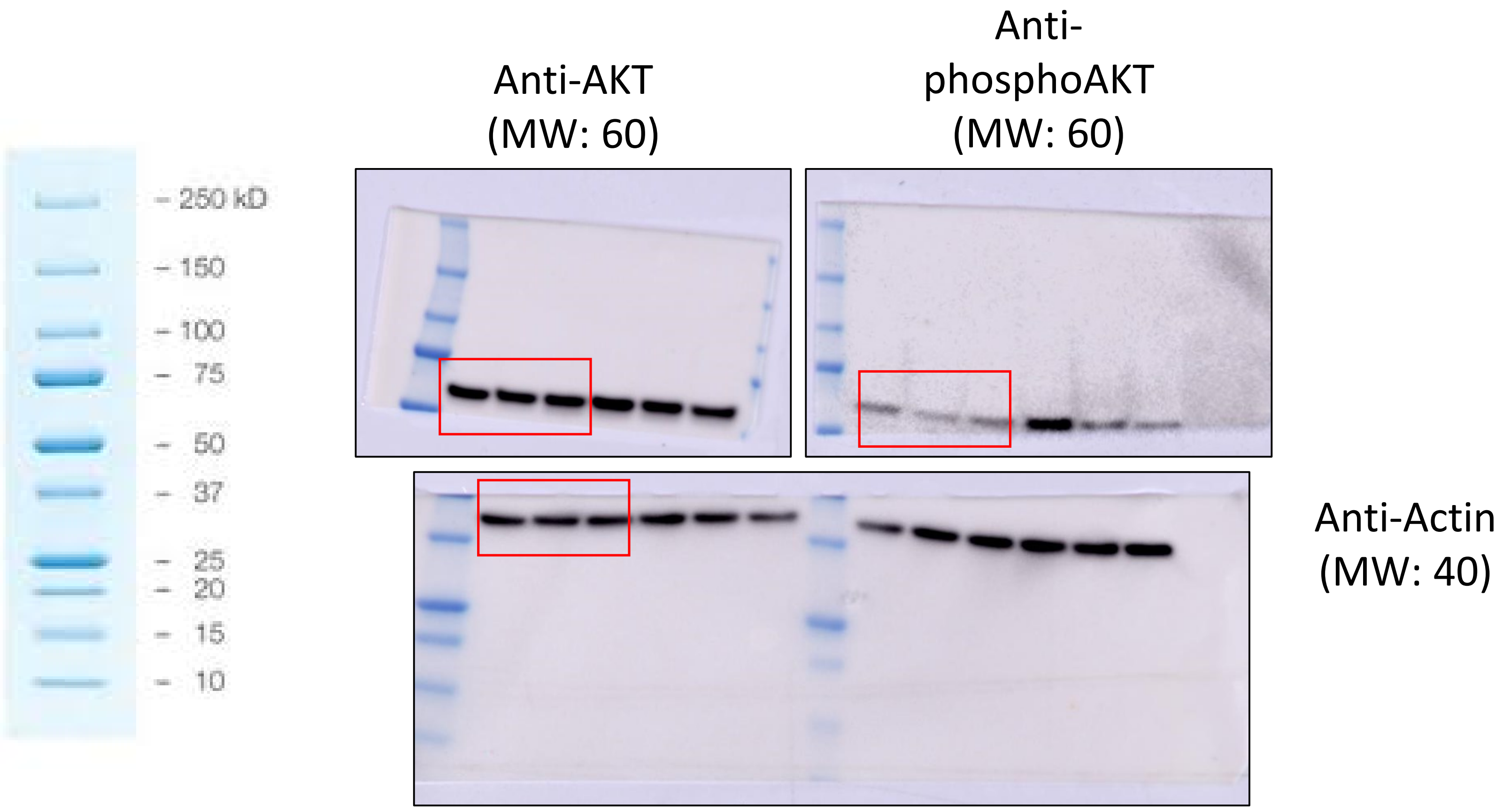

Anti-AKT
(MW: 60)
Anti-
phosphoAKT
(MW: 60)
Anti-Actin
(MW: 40)
250 kD
150
100
75
50
37
25
20
15
10

Fig. 4I

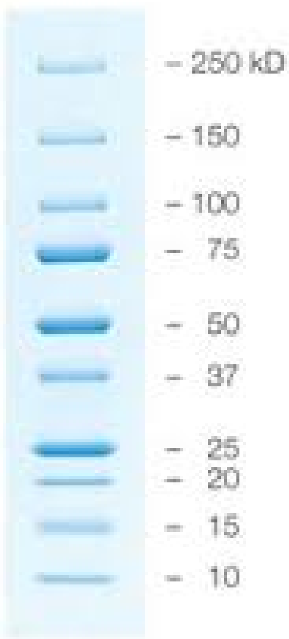

Anti-phosphoS6K1
(MW: 70)

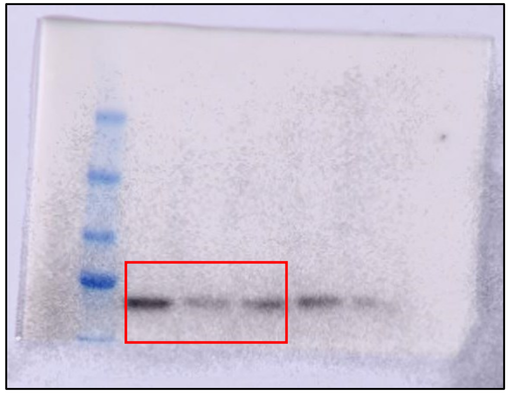

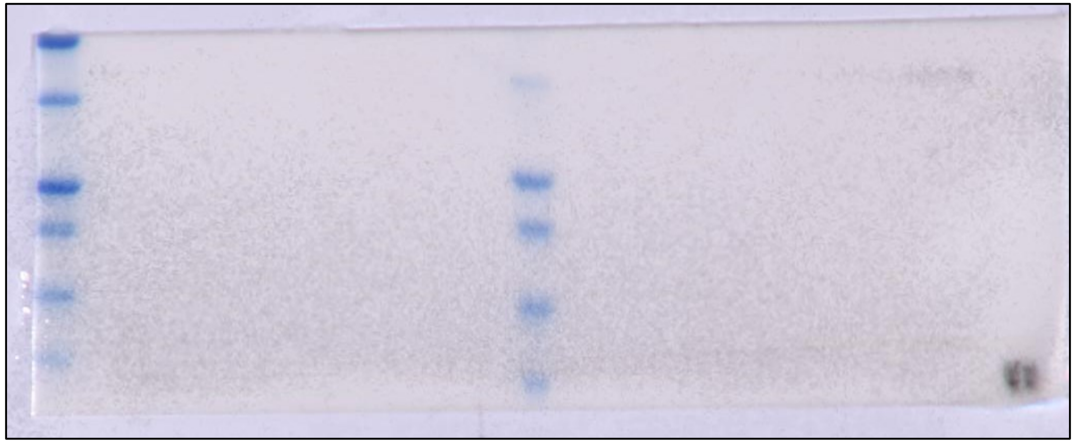

Anti-S6K1
(MW: 70)

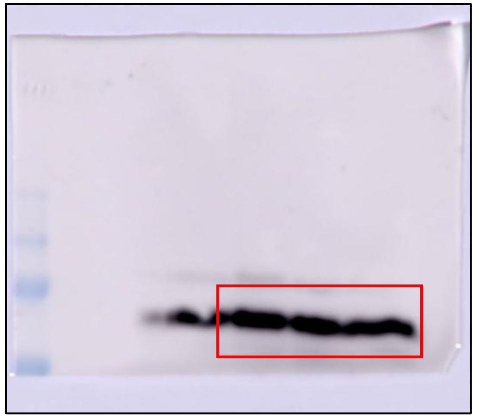

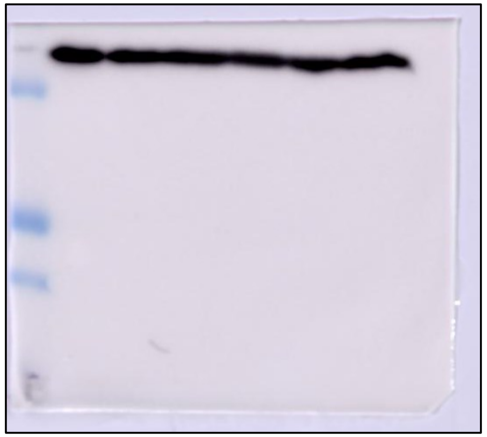

Anti-Actin (MW: 40)
not shown in the paper

Fig. 4M

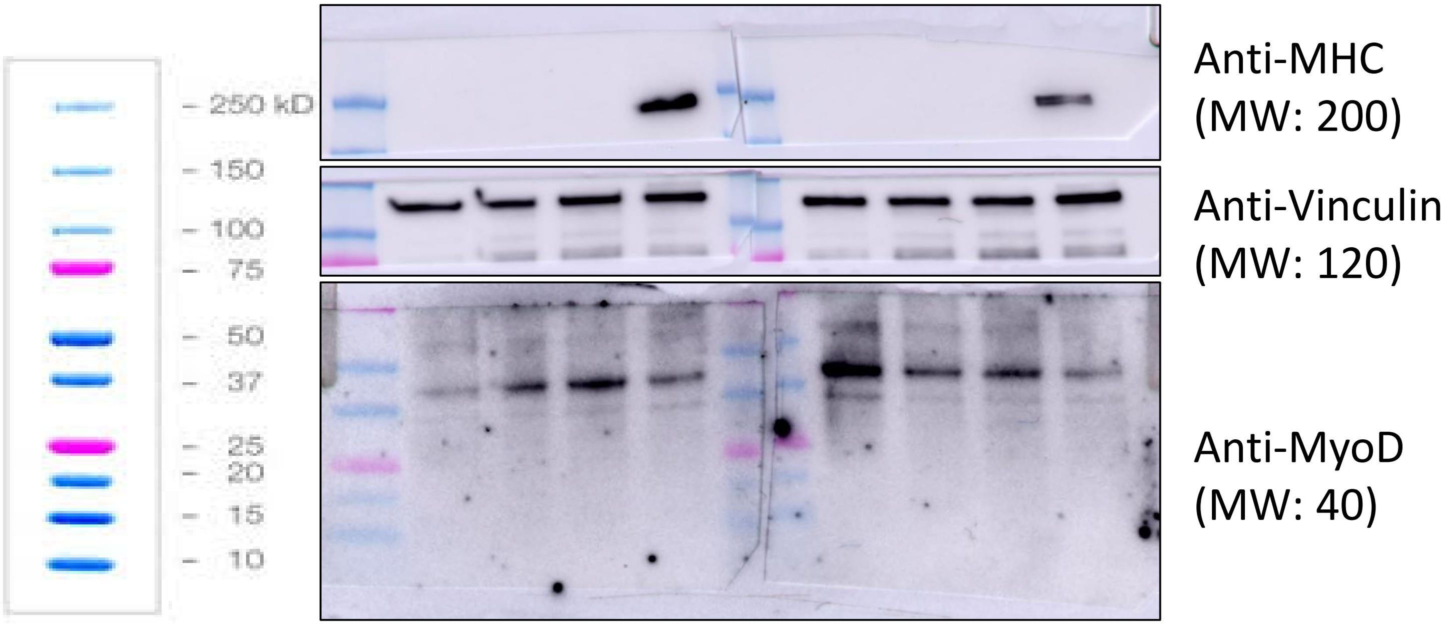

250 kD
150
100
75
50
37
25
20
15
10
Anti-MHC
(MW: 200)
Anti-Vinculin
(MW: 120)
Anti-MyoD
(MW: 40)

Fig. 5H

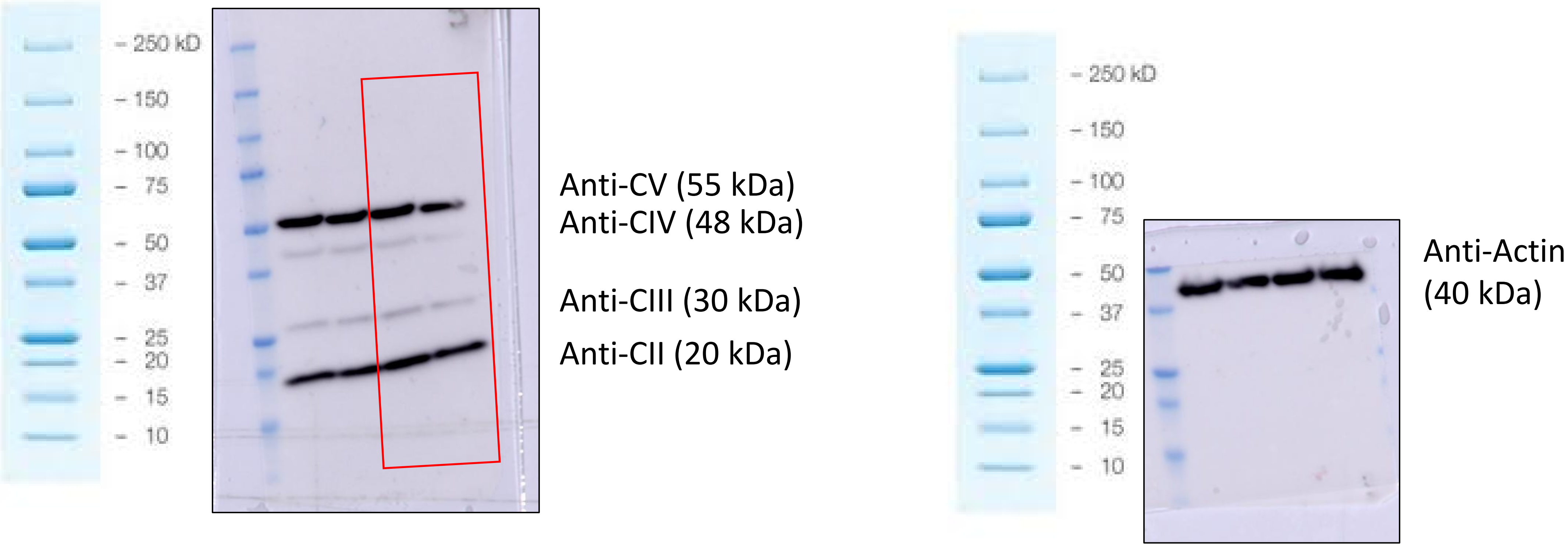
250 kD
150
100
75
50
37
25
20
15
10
Anti-CV (55 kDa)
Anti-CIV (48 kDa)
Anti-CIII (30 kDa)
Anti-CII (20 kDa)
250 kD
150
100
75
50
37
25
20
15
10
Anti-Actin
(40 kDa)
